# Testisin/*Prss21* deficiency causes increased vascular permeability and a hemorrhagic phenotype during luteal angiogenesis

**DOI:** 10.1371/journal.pone.0234407

**Published:** 2020-06-08

**Authors:** Raymond J. Peroutka, Marguerite S. Buzza, Subhradip Mukhopadhyay, Tierra A. Johnson, Kathryn H. Driesbaugh, Toni M. Antalis

**Affiliations:** 1 Department of Physiology, Center for Vascular and Inflammatory Diseases, University of Maryland School of Medicine, Baltimore, Maryland, United Sates of America; 2 VA Maryland Health Care System, Baltimore, Maryland, United Sates of America; 3 Marlene and Stewart Greenebaum Comprehensive Cancer Center, University of Maryland School of Medicine, Baltimore, Maryland, United Sates of America; 4 Department of Surgery, University of Maryland School of Medicine, Baltimore, Maryland, United Sates of America; University of Illinois at Chicago, UNITED STATES

## Abstract

Testisin (encoded by *PRSS21*) is a membrane anchored serine protease, which is tethered to the cell surface via a glycosylphosphatidylinositol (GPI)-anchor. While testisin is found in abundance in spermatozoa, it is also expressed in microvascular endothelial cells where its function is unknown. Here we identify testisin as a novel regulator of physiological hormone-induced angiogenesis and microvascular endothelial permeability. Using a murine model of rapid physiological angiogenesis during corpus luteal development in the ovary, we found that mice genetically deficient in testisin (*Prss21*^*-/-*^) show a substantially increased incidence of hemorrhages which are significantly more severe than in littermate control *Prss21*^*+/+*^ mice. This phenotype was associated with increased vascular leakiness, demonstrated by a greater accumulation of extravasated Evans blue dye in *Prss21*^*-/-*^ ovaries. Live cell imaging of *in vitro* cultured microvascular endothelial cells depleted of testisin by siRNA knockdown revealed that loss of testisin markedly impaired reorganization and tubule-like formation on Matrigel basement membranes. Moreover testisin siRNA knockdown increased the paracellular permeability to FITC-albumin across endothelial cell monolayers, which was associated with decreased expression of the adherens junction protein VE-cadherin and increased levels of phospho(Tyr658)-VE-cadherin, without affecting the levels of the tight junction proteins occludin and claudin-5, or ZO-1. Decreased expression of VE-cadherin in the neovasculature of *Prss21*^*-/-*^ ovaries was also observed without marked differences in endothelial cell content, vascular claudin-5 expression or pericyte recruitment. Together, these data identify testisin as a novel regulator of VE-cadherin adhesions during angiogenesis and indicate a potential new target for regulating neovascular integrity and associated pathologies.

## Introduction

The endothelium plays a critical role in regulating vascular wall functions, such as modulating vascular tone, controlling the exchange of fluids and cells, regulating local cellular growth and extracellular matrix deposition, and controlling homeostatic as well as inflammatory responses [1]. The endothelium is also the site of angiogenesis, the multistep process of vascular remodeling involving coordinated migration, proliferation, and junction formation of vascular endothelial cells to form new vessel branches in response to growth stimuli [2]. Endothelial cells constitute virtually the entirety of newly formed small microvessels or capillaries, which are stabilized through further maturation that includes reconstitution of the basement membrane and the recruitment of smooth muscle cells/pericytes that encircle the endothelial tubule [3]. Unresolved vascular remodeling and endothelial dysfunction promote vascular permeability and inflammation, a feature of many pathological states and diseases, including coronary artery disease, atherosclerosis, hypertension, diabetes and tumor metastasis [4].

Intercellular junctions between endothelial cells mediate barrier integrity and control barrier permeability [5]. There are three major subtypes of intercellular junctions; tight junctions (TJ), adherens junctions (AJ), and gap junctions (GJ) [6, 7]. The cell-cell interactions between endothelial cells are mediated by the AJ protein, vascular endothelial (VE)-cadherin, which is specifically responsible for endothelial AJ assembly and barrier architecture [8], and dictates the levels of expression and localization of other junctional molecules including claudin-5 and N-cadherin [9, 10]. Several lines of evidence demonstrate that VE-cadherin plays a pivotal role in angiogenesis and controls vascular permeability [11].

Testisin (*PRSS21*) is a membrane-anchored trypsin-like serine protease, which is synthesized with a distinct carboxy-terminal peptide that is post-transcriptionally modified with a glycosylphosphatidylinositol (GPI)-membrane anchor [12–15]. Testisin is abundantly expressed in testis [13, 16, 17] and genetic ablation of testisin in mice results in developmentally damaged spermatocytes and decreased fertilizing ability [17–19]. Testisin deficient sperm exhibit morphological defects including angulated and curled tails, and fragile necks, resulting in increased decapitation and decreased motility [17]. While testisin shows limited expression or is not expressed in most other tissues [13, 14, 20–23], we showed that testisin mRNA is expressed by human microvascular endothelial cells (HMVECs) undergoing reorganization and tube-like formation in Matrigel angiogenesis assays *in vitro* and during pre-capillary morphogenesis on 3-D fibrillar type I collagen [12]. Separately, a global analysis of human endothelial cell diversity, utilizing microarray analysis of 14 distinct vascular tissues, identified testisin as expressed consistently in both large vessels and the microvasculature [24]. However, whether testisin plays a functional role in angiogenesis is not known.

Unlike secreted serine proteases, which function primarily in tissue repair, immunity, and nutrient uptake, the membrane-anchored serine proteases typically are found to regulate fundamental cellular and developmental processes, such as tissue morphogenesis, epithelial barrier function, ion and water transport, cellular iron export, and fertilization [25]. In addition, membrane-anchored serine proteases are also known to activate growth factors and other protease zymogens that induce cell proliferation, extracellular matrix remodeling and cell migration [26]. Anchored directly to the plasma membrane, testisin is not only an integral component of the cell surface but its GPI-anchor further restricts its localization to cholesterol rich regions of the membrane, known as lipid rafts [27]. Thus testisin proteolytic activity in endothelial cells could be predicted to function in multiple biological processes that are implicated in the formation of a functional blood vessel, including basement membrane remodeling, endothelial migration and extracellular matrix invasion, capillary tubule development and lumen formation during angiogenesis [28, 29].

Here we have investigated the functional role of testisin in a murine model of rapid physiological angiogenesis associated with corpus luteum development after gonadotropin-stimulated ovulation. We find that *Prss21*^*-/-*^ deficiency results in the significant development of multiple hemorrhagic corpora lutea that are associated with vascular leakage. Cell based assays utilizing siRNA knockdown of testisin in endothelial cells show that testisin is required for angiogenic endothelial cell tube-like formation on Matrigel basement membranes, and for maintaining endothelial barrier function. The reduction or absence of testisin expression, *in vitro* and *in vivo*, was associated with significant decreases in both expression of VE-cadherin and its localization at intercellular junctions, establishing for the first time a novel functional role for a membrane anchored serine protease in junctional remodeling during angiogenesis.

## Materials and methods

### Animals

*Prss21*^*-/-*^ knockout mice on the C57BL/6 genetic background were generated from Prss21^tm1a(KOMP)Wtsi^ embryonic stem cells, obtained from the Knockout Mouse Project (www.KOMP.org), as detailed in S1 Fig. Mice were weaned at 3 weeks of age, maintained on a 12-hour light/dark cycle, and fed a standard rodent diet. Studies were performed using age-matched littermates generated through heterozygous crosses. All animal studies were performed in compliance with the University of Maryland School of Medicine Office of Animal Welfare Assurance Guidelines for Animal Research and protocols were approved by the University of Maryland School of Medicine Institutional Animal Care and Use Committee (IACUC).

### Luteal angiogenesis assay and characterization of hemorrhage

Immature female *Prss21*^*+/+*^ and *Prss21*^*-/-*^ mice (age 23–26 days post-natal) were superovulated by i.p. injection of 5 IU of pregnant mare serum gonadotropin (PMSG)(Sigma) followed 48 hours later with 5 IU human chorionic gonadotropin (hCG)(Sigma). At empirically determined times between 12 and 96 hours post-hCG injection, animals were weighed, euthanized, and the ovaries removed, trimmed and weighed. The ovaries were visually inspected and whole mounts photographed using a Leica EZ4W. The ovaries were then either fixed or frozen for histological and molecular analyses. The incidence of hemorrhage was quantified by counting the number of hemorrhages greater than approximately 100 μm in diameter which were readily visible on each ovary. Each hemorrhage was graded according to a visual scale of 1–4 with assignments based on the following diameter range: grade 1 (100–200 μm), grade 2 (200–300 μm), grade 3 (300–600 μm), grade 4 (>600 μm). The severity of hemorrhages per ovary represents the sum of the grades per ovary as an approximate measure of total hemorrhage burden. Internal hemorrhages found during subsequent histological examination were not retroactively included. The incidence and severity of hemorrhage was assessed independently by at least two investigators blinded to genotype.

### Antibodies

The anti-testisin monoclonal antibody MAbD9.1 was purified from the PTA-6077 hybridoma cell line (ATCC Pro104.D9.1) and characterized for both mouse and human anti-testisin specificity (S2 Fig), since available commercial anti-testisin antibodies were found not to specifically recognize murine or human testisin. Additional antibodies were rat anti-CD31 (BD 553370), rabbit anti-occludin (Invitrogen 71–1500), rabbit anti-ZO-1 (Invitrogen 61–7300), rabbit anti-claudin-5 (Invitrogen 34–1600), rabbit anti-NG-2 (Millipore AB5320), mouse anti-VE-cadherin (Santa Cruz sc-9989), rabbit anti-VE-cadherin (Abcam ab33168), rabbit anti-phospho (Tyr658)-VE-cadherin (Invitrogen 44-1144G), rabbit anti-β-catenin (Cell Signaling 8480), rabbit anti-GAPDH (Cell Signaling 2118), rabbit anti-β-actin (Cell Signaling 4970). Secondary antibodies were goat anti-mouse-HRP (Jackson ImmunoResearch 115-035-146), mouse anti-rabbit-HRP (Jackson ImmunoResearch 211-035-109), goat anti-rat-Alexa Fluor 488 (Invitrogen A11006), goat anti-mouse-Alexa Fluor 555 (Invitrogen A32727), goat anti-rabbit-Alexa Fluor 555 (Invitrogen A32732), goat anti-mouse-Alexa Fluor 488 (Invitrogen A32723).

### Cell culture

HMEC-1 cells (CRL-3243) were purchased from American Type Culture Collection and maintained in complete MCDB-131 (Gibco) medium supplemented with 10 ng/mL epidermal growth factor (Peprotec),1 μg/mL hydrocortisone (Sigma), 2 mM GlutaMax (Gibco), and 10% fetal bovine serum (Sigma). HMVEC-d cells (FC-0042) were purchased from Lifeline Cell Technology. HUVEC cells (C0155C) were purchased from Gibco. HMVEC-d and HUVEC cells were maintained in complete VascuLife VEGF-Mv media (LL-0005) from Lifeline Cell Technology and cultured on surfaces coated with 0.1% gelatin (Sigma). HMEC-1 cells were used between passage 3–10 while HMVEC-d and HUVEC cells were used between passage 3–8. Cells were routinely tested for mycoplasma using MycoAlert (Lonza).

### siRNA knockdown

HMEC-1 endothelial cells were transfected with testisin Silencer Select siRNAs (s223167; denoted siTs67) and (s223168; denoted siTs68), testisin Stealth RNAi (HSS116894; denoted siTs94) and control non-target siRNA (4390843; denoted siNC) (ThermoFisher). The transfection conditions for silencing testisin mRNA and protein expression while ensuring cell viability were optimized (S3 Fig), and two siRNAs (siTs67 and siTs94) were identified for experimental use. Transfections were carried out with 5–15 nM siRNA and DharmaFECT 1 transfection reagent (Dharmacon). Cell viability was monitored using PrestoBlue (ThermoFisher).

### RNA isolation and quantitative real-time PCR (qPCR)

RNA was isolated from cultured endothelial cells using the PureLink RNA mini kit (ThermoFisher). For animal studies, tissue was collected, flash frozen, and RNA isolated using the RNeasy Plus Universal kit (Qiagen). cDNA was generated by reverse transcription with the High Capacity Reverse Transcription kit (ThermoFisher). TaqMan Master Mix qPCR was used for target amplification and reactions run using either the QuantStudio 3 (ThermoFisher) or 7900HT PCR system (Applied Biosystems). qPCR values for samples were normalized to GAPDH and expressed as fold change relative to a control sample (ΔΔCt) or as the fold change relative to GAPDH (ΔCt), as indicated in the figure legend. Mouse and human specific TaqMan Primers were purchased from ThermoFisher: GAPDH (Hs99999905_m1), testisin (Hs00199035 and Mm00480386), CD31 (Mm01242576), CD105 (Mm00468252), and VEGFA (Hs00900055, Ms00437306).

### Endothelial cell tubule formation assay

Culture dishes (24 well) were coated with 200 μl/well of 10 mg/ml Matrigel (Becton Dickinson) on ice, and Matrigel was allowed to solidify for 30 min at 37°C. Seventy-two hours post-transfection, HMEC-1 and testisin siRNA depleted HMEC-1 cells (7.5x10^4^) in 500 μl of complete MCDB-131 were seeded into each Matrigel-coated well and allowed to settle for ~10 min. Endothelial cell tubules were allowed to form at 37°C in an environmentally controlled incubator (5% CO_2_), and cell morphology changes and tubular-structure formation were imaged automatically every 20 minutes by phase contrast microscopy using an EVOS FL Auto (Life Technologies) for up to 24 hours. The degree of tube formation (total tube length) was quantified by image analysis using the Angiogenesis Analyzer software plugin [30] for NIH ImageJ. In some experiments, RNA was collected from Matrigel wells using an equal volume of Trizol (Invitrogen) and purified as per the manufacturer’s instructions. Cell viability was measured by PrestoBlue assay. Each experiment was performed with triplicate wells per experimental condition.

### Histopathology and immunohistochemistry

Ovaries were fixed in zinc fixative (BD Pharmingen) overnight and then stored in 70% ethanol. The specimens were embedded in paraffin and 5 μm deparaffinized sections stained with hematoxylin and eosin (H&E) or subjected to immunohistochemical analyses. Heat induced antigen retrieval was performed on all sections using citrate buffer pH 6.0. After antigen retrieval, sections were washed twice in TBS, blocked for 1 hour at room temperature with 10% goat serum + 1% BSA in TBS and incubated overnight at 4°C with primary antibody diluted in 1% BSA in TBS. Hydrogen peroxidase blocking with 0.3% H_2_O_2_ in TBS was performed after primary antibody incubation. Sections were washed twice and incubated with biotinylated secondary antibody for 1 hour at room temperature. Detection of specific antigens was performed by development with Vectastain Elite ABC Kit (Vector Laboratories). Sections were incubated with diaminobenzidine (DAB) substrate-chromogen solution, and slides were counterstained with hematoxylin prior to being dehydrated and mounted with Permount (ThermoFisher). Images of slides were obtained by the EVOS FL Auto Cell Imaging System (Life Technologies).

### *In vitro* permeability assay

HMEC-1 cells were transfected with siRNAs and 24 hours post transfection, cells were re-plated (2.5x10^5^ per insert in 500 μl of complete media) in the upper chamber of 24-well (12 mm) 0.4 μm transwell inserts (Corning). A hydrostatic volume of complete media (1.5 ml) was added to the lower chamber. The next day non-adherent cells were removed from the transwell insert by washing with PBS and fresh complete media added. At 72 hours post transfection, the paracellular flux of albumin across the endothelial monolayers was assayed by adding FITC-albumin (1 mg/ml, Sigma) to the apical compartment and the assay incubated for 1 hour at 37°C. Media was removed from the lower chamber and fluorescence was measured (ex495nm/em520nm) using a FlexStation 3 (Molecular Devices). Fluorescence data is represented as signal relative to the negative control siRNA. Cell viability was monitored using PrestoBlue (1:20 dilution, Invitrogen) added to the apical chamber and fluorescence measured (ex560nm/em590nm). In some experiments the transwell inserts of replicate wells were fixed in 4% paraformaldehyde at room temperature and immunostained for detection of VE-cadherin and β-catenin by fluorescence microscopy. Sections were counterstained with DAPI for detection of nuclei. Confocal images were captured using an LSM 800 confocal microscope (ZEISS). Images captured using the EVOS FL Auto Cell Imaging System (Life Technologies) were analyzed using ImageJ.

### Immunoblotting

Endothelial cells were solubilized using either ice-cold lysis buffer (50mM HEPES pH 7.3, 150 mM NaCl, 0.5% Triton, and 0.5% NP40) or directly in 1x LDS loading buffer (Invitrogen) with 2-mercaptoethanol (βME, Sigma). Lysis buffers were prepared with Complete Mini Protease Inhibitor Cocktail & PhosSTOP Phosphatase Inhibitor Cocktail (Roche). Samples were sonicated briefly, spun, and in the case of samples in lysis buffer, protein concentrations determined by Bradford assay. Lysis buffer samples, normalized for protein concentration, were mixed with 4x LDS loading buffer with βME. Samples were heated to 70°C for 10 minutes prior to loading. Samples were resolved on either denaturing 12% or 4–12% Bis-Tris NuPage gels (Invitrogen). Proteins were transferred to Immobilon P PVDF (Millipore), blocked with either 5% non-fat milk or 5% BSA in PBS (pH 7.5) and typically incubated with primary antibody in PBST (PBS + 0.05% tween-20) and 5% BSA overnight at 4°C, and HRP-conjugated secondary in PBST with 5% non-fat milk for 1 hour the next day. HRP conjugates were detected with SuperSignal West Pico chemiluminescent substrate (Pierce). Blots were exposed using either Amersham Hyperfilm ECL (GE Life Sciences) or HyBlot CL (Denville Scientific) and densitometry analysis was performed using ImageJ. Density was calculated by subtracting background and dividing by area. All areas compared were of equal size, while multiple exposures were developed to ensure that none of the bands compared had reached saturation.

### Analysis of vascular permeability by Evans blue dye extravasation

At 24 hours post hCG, superovulated female *Prss21*^*+/+*^ and *Prss21*^*-/-*^ mice were anesthetized and retro-orbital injections performed using a sterile 1% solution of Evans blue dye in normal saline (~80 mg/kg of body weight) as described [31]. After 30 min, mice were euthanized, and vessels cleared by transcardial perfusion with 20–30 ml of ice-cold PBS, 2 mM EDTA. Ovaries were removed, trimmed and photographed. The ovaries were then weighed and either frozen in liquid nitrogen and stored at −80°C or preserved in OCT for histological analyses. Evans blue permeation into tissues was quantitated by homogenization of frozen ovaries in a 1:3 (mg/ml) volume of 50% trichloroacetic acid (TCA), brief sonication and centrifugation (30 min, 15,000 rcf, 4°C) [32]. Evans blue dye content in the supernatant was measured by fluorescence (ex620/em680) and quantified according to a standard curve. The results are presented as (μg of Evans blue dye)/(g of tissue).

### Immunofluorescence staining of tissues and quantitative analyses

Collected ovaries were immediately embedded in Tissue-Tek OCT Compound (Sakura Finetek USA), frozen in a pre-chilled isopentane dry ice bath, and stored at -80° until sections (7μm) were prepared and mounted on Superfrost Plus slides (ThermoFisher). Slides were fixed in ice-cold acetone for 10 mins, dried at room temperature and washed with wash buffer (PBS + 1% BSA + 0.2% Tween). Samples were blocked in wash buffer with 2% goat serum (Sigma) for 30 min and incubated with primary antibodies overnight at 4°C. Following washing the next day, secondary Alexa Fluor conjugated secondary antibodies were incubated for one hour at room temperature. Sections were counterstained with 0.5ug/ml DAPI and mounted using ProLong Glass (Invitrogen).

CD31 positive staining in endothelial cells was quantitated using Image J. To quantitate VE-cadherin and claudin-5 positive endothelial cells separate from background staining in granulosa cells, CD31 positive cells were initially selected on images of co-stained sections using the threshold command. The selection was then inversed and used to remove non-endothelial staining from the co-stained channel (VE-cadherin or claudin-5). ImageJ was then used to define the corpora lutea regions using the freehand tool, apply defined threshold values, and quantitate fluorescent intensity values. Tissue sections containing at least three distinct corpora lutea were analyzed from at least three mice per genotype to determine mean fluorescent values. All images presented are originals.

### Statistical analysis

Data are presented as the mean ± SEM. Comparison of experimental groups was performed by either an unpaired *t-*test or one-way repeated analysis of variance (ANOVA). ANOVA analysis was followed by a Tukey's multiple comparisons test to determine the differences between specific groups. Data was analyzed utilizing GraphPad Prism 8.0 software, p values < 0.05 were considered statistically significant.

## Results

### Testisin knockout mice display a pronounced hemorrhagic phenotype in ovaries after gonadotropin-induced ovulation

**Fig 1:**
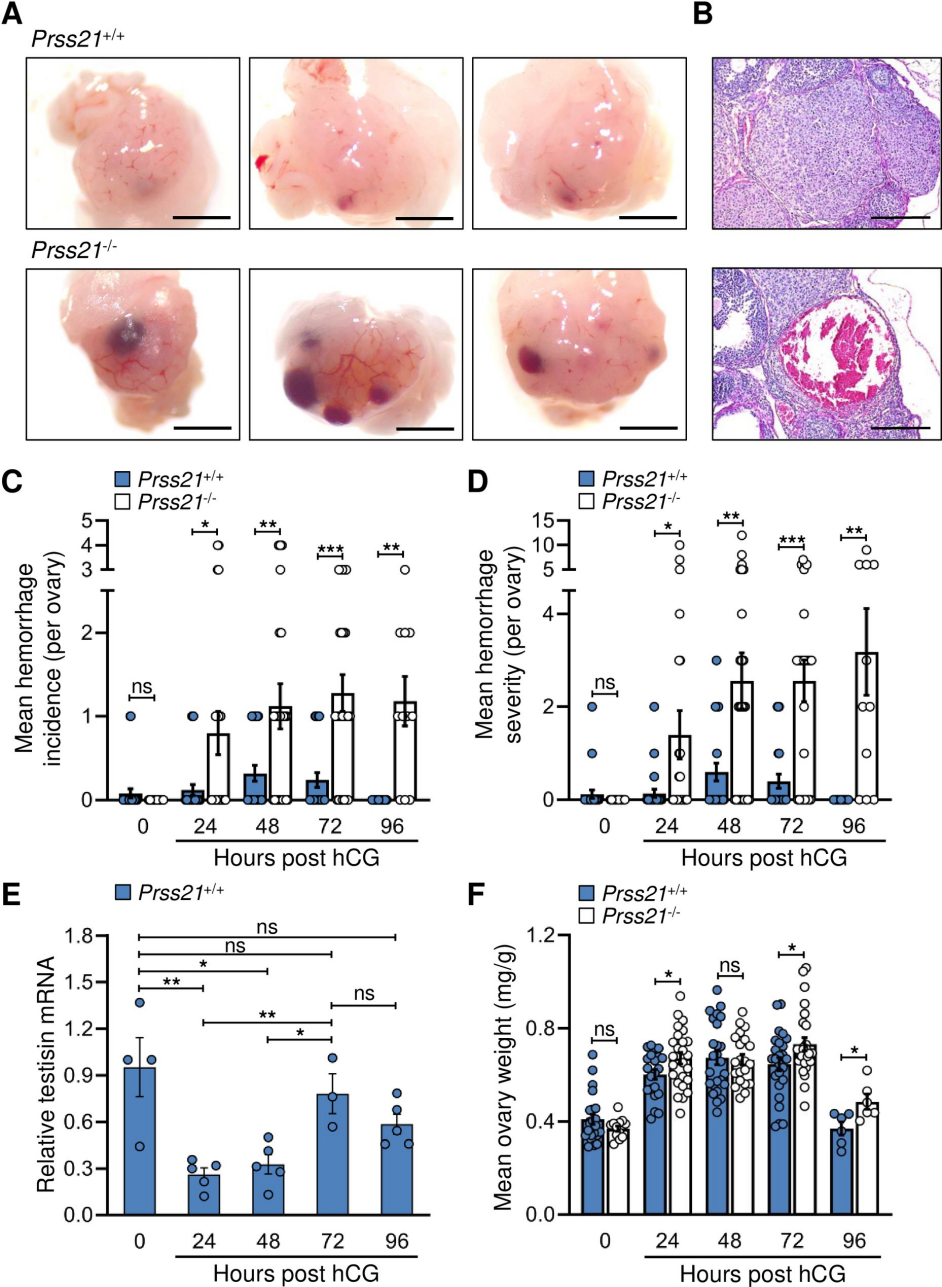
*Prss21*^*-/-*^ mice display a hemorrhagic phenotype during vascularization of the corpus luteum. **A)** Physiological angiogenesis in the ovaries of *Prss21*^*+/+*^ and *Prss21*^*-/-*^ mice was initiated by administration of PMSG and hCG to induce ovulation and vascularization of the corpus luteum. Images are of representative whole mount ovaries at 72h post hCG injection showing multiple large hemorrhages in *Prss21*^*-/-*^ as compared to *Prss21*^*+/+*^ ovaries. Original magnification, 16.5x. Scale bar = 1000 μm. **B)** Representative H&E stained sections at 72h post hCG injection showing a large hemorrhage in the corpus luteum of a *Prss21*^*-/-*^ ovary as compared to a *Prss21*^*+/+*^ ovary. Original magnification, 20x. Scale bar = 200 μm. **C)** *Prss21*^*-/-*^, as compared to *Prss21*^*+/+*^, whole mount ovaries demonstrated significantly increased incidences of hemorrhages at 24, 48, 72 and 96 hours post hCG by visual inspection (0h n = 22–25, 24h n = 25, 48h n = 25, 72h n = 25, 96h n = 10–11, where n = number of ovaries per genotype). **D)** *Prss21*^*-/-*^, as compared to *Prss21*^*+/+*^, whole mount ovaries also demonstrated a significant increase in the severity of hemorrhages at 24, 48, 72 and 96 hours post hCG (0h n = 22–25, 24h n = 25, 48h n = 25, 72h n = 25, 96h n = 10–11, where n = number of ovaries per genotype). The hemorrhage severity score as a measure of hemorrhage burden was determined as defined in the methods. **E)** qPCR analysis of testisin mRNA expression in whole ovary homogenates over the time course of luteal angiogenesis. (0h n = 4, 24h n = 5, 48h n = 5, 72h n = 4, 96h n = 2, where n = number of ovaries). **F)** Weight of ovaries, normalized to body weight, from testisin knockout mice were significantly higher compared to wild type controls at 24, 72 and 96 hours post hCG (0h n = 13–23, 24h n = 21–25, 48h n = 21–25, 72h n = 25, 96h n = 6–6, where n = number of ovaries per genotype). Mean ± SEM. ***: p<0.001, **: p<0.01, *: p<0.05, ns: not significant.

Angiogenesis is a critical component of normal follicular and luteal function in the ovary. Following ovulation, the developing corpus luteum (CL) is a site of rapid and intense angiogenesis. The thecal-based endothelial cells both invade and proliferate to form a dense vascular network which provide the newly steroidogenic granulosa cells with hormone precursors, nutrients, and oxygen. This process results in the synthesis and release the large amounts of hormones, predominantly progesterone, which are required for implantation and maintenance of early pregnancy [33]. To investigate the potential role of testisin during this rapid angiogenic response *in vivo*, *Prss21*^*+/+*^ and *Prss21*^*-/-*^ mice were generated (S1 Fig). The *Prss21*^*-/-*^ mice show normal female fertility and Mendelian inheritance, consistent with previously published data [17, 18]. The effect of testisin genetic deficiency on angiogenesis was examined by comparing ovaries of *Prss21*^*+/+*^ and *Prss21*^*-/-*^ mice after gonadotropin-induced ovulation. Ovulation was induced in immature female *Prss21*^*+/+*^ and *Prss21*^*-/-*^ mice by administration of PMSG followed 48 hours later by hCG. Development of the corpus luteum and progesterone production occurs naturally in this model, reaching peak progesterone levels at ~72 hours post hCG administration, which in the absence of pregnancy is followed by luteolysis and vascular regression [33]. Comparison of ovaries from female *Prss21*^*+/+*^ and *Prss21*^*-/-*^ mice by visual inspection at 72 hours post hCG administration revealed multiple prominent blood pools present in the *Prss21*^*-/-*^ mouse ovaries as compared to the ovaries of the *Prss21*^*+/+*^ littermate controls (Fig 1A). Although some bleeding can occur following rupture of the mature ovarian follicle (referred to as a corpus hemorrhagicum), hematoxylin and eosin stained histological sections demonstrated the presence of multiple blood-filled corpora lutea prevalent in the *Prss21*^*-/-*^ ovaries (example in Fig 1B).

The number of ovarian hemorrhages and hemorrhage severity were quantitated at 0 and post hCG injection 24, 48 and 72 hours. The incidence of hemorrhages observed in the ovaries of *Prss21*^*+/+*^ mice over the time course was low, with most ovaries showing no or a few small hemorrhages, and none at 96 hours. In contrast, by 48 hours post hormonal treatment, the ovaries of *Prss21*^*-/-*^ mice averaged at least one hemorrhage per ovary (Fig 1C), with as many as four in some ovaries. The presence of hemorrhages in ovaries of *Prss21*^*-/-*^ mice persisted throughout the 96 hour post hCG time course, when hemorrhages in *Prss21*^*+/+*^ ovaries could no longer be detected. Grading of hemorrhage size per ovary as a measure of hemorrhage severity revealed significantly larger and more severe hemorrhages in the ovaries of *Prss21*^*-/-*^ mice compared to *Prss21*^*+/+*^ ovaries (Fig 1D).

Analysis of testisin expression over the time course of corpus luteal development showed that testisin mRNA is highest in the immature ovary and decreases substantially by 24 hours (Fig 1E). This drop in testisin expression coincides with permeability increases that are consistently observed in the beginning of angiogenesis [34, 35]. As vascularization of the corpus luteum proceeds, testisin mRNA levels rise dramatically, reaching a maximum at 72 hours post hCG administration, correlating with maximal vascularization and progesterone levels [33], following which there is a subsequent drop, in line with luteolysis and vascular regression. During development of the corpus luteum, ovary weights increased to a maximal 1.6-fold in *Prss21*^*+/+*^ mice between 48 to 72 hours compared to non-hormonally treated immature ovaries at 0 hours and returned to normal weights after 96 hours (Fig 1F). In contrast, the ovary weights of *Prss21*^*-/-*^ mice significantly increased to the maximal *Prss21*^*+/+*^ levels by 24 hours and remained significantly elevated at both 72 and 96 hours compared to *Prss21*^*+/+*^ ovaries (Fig 1E), possibly indicative of edema. Together, these data show that testisin expression is modulated during hormonally induced ovulation in mice and its expression correlates with vascularization of the corpus luteum.

### Testisin is expressed in endothelial cells and is upregulated during microvascular endothelial tubule-like formation on Matrigel

**Fig 2:**
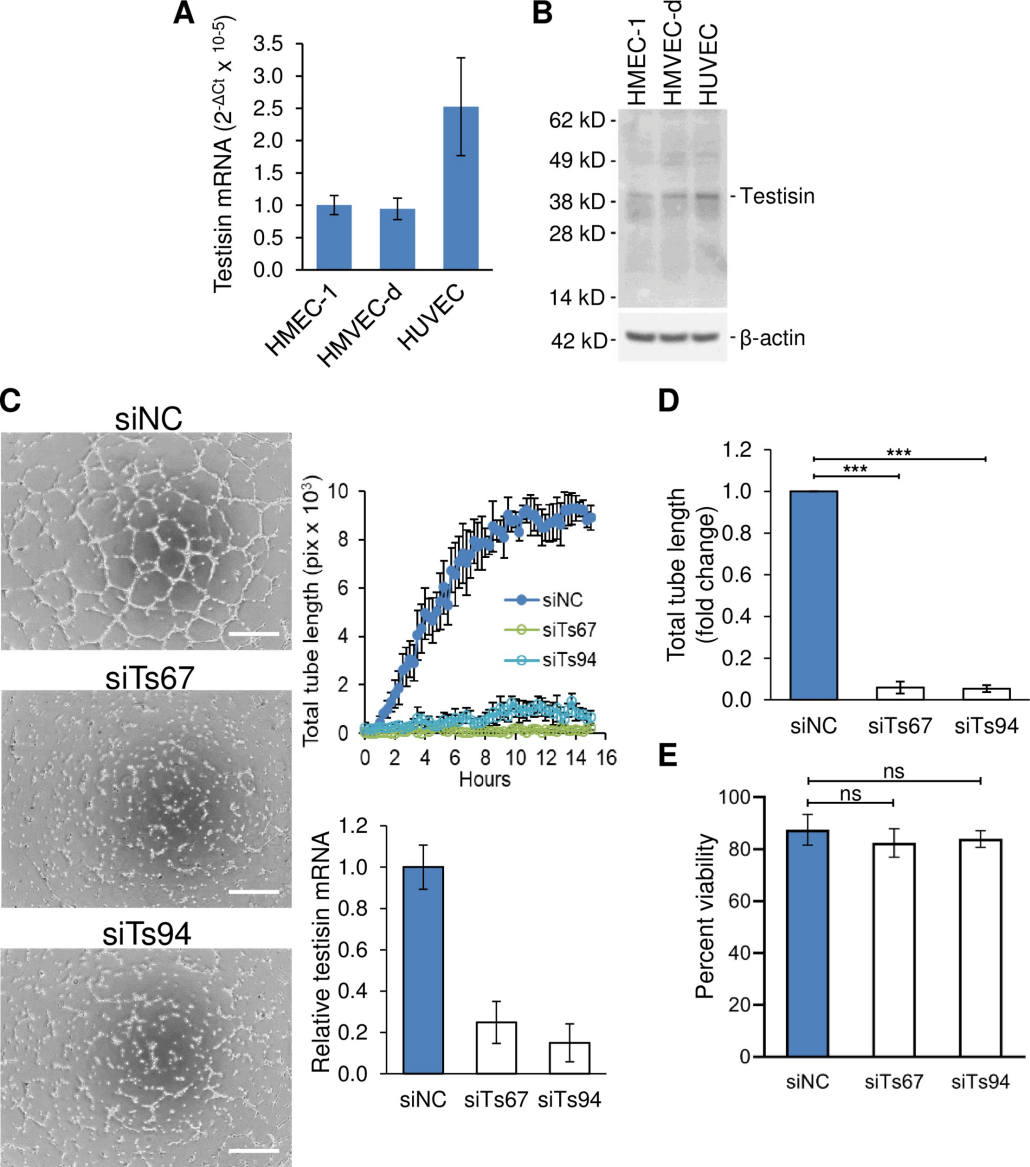
Testisin is expressed in endothelial cells and its silencing inhibits HMEC-1 reorganization and tubule-like formation on Matrigel. **A)** Total RNA from confluent HMEC-1, HMVEC-d and HUVEC cells was isolated and analyzed by qPCR for testisin expression. mRNA levels are relative to GAPDH mRNA and expressed using the 2^-ΔCt^ method. Graph shows mean ± SEM from three independent experiments. **B)** Immunoblot analysis of testisin protein expression relative to β-actin in lysates (total protein 50 μg) prepared from HMEC-1, HMVEC-d and HUVEC cells. Data is representative of two independent experiments. **C)** Disruption of tubule formation after testisin silencing. (*left*) Representative images of control (siNC) and testisin siRNA (siTs67, siTs94) transfected HMEC-1 cells are shown after 15 hours incubation on polymerized Matrigel, taken at 4x magnification. Scale bar = 500 μm. (*right*, *top*) Time course of endothelial cell tubule formation. Quantitation of total tube length from images automatically taken every 20 minutes during the 15-hour incubation. Error represents ± SD from triplicate wells. (*right*, *bottom*) At the end of the experiment, testisin siRNA knockdown was verified by qPCR analysis of mRNA isolated from the polymerized Matrigel. Graph shows mean ± SD from triplicate wells. **D)** Endpoint analysis of total tube length demonstrated a significant difference between testisin knockdown and control endothelial cells. Signals are expressed as relative to siNC. The graph shows the mean ± SEM from 4 independent experiments, each an average of triplicate wells. **E**) Cell viability was unaffected by testisin knockdown as measured by PrestoBlue assay at the end of the experiments. Signals are expressed as relative to siNC. Graphs show mean ± SEM and represents the mean of four independent experiments. ***: p<0.001, **: p<0.01, *: p<0.05, ns: not significant.

Testisin expression was analyzed in three endothelial cell types cultured *in vitro*: primary human dermal microvascular endothelial cells (HMVEC-d), the immortalized derivative of HMVEC-d (HMEC-1 cells) and primary human umbilical vein cells (HUVEC). All three endothelial cell types were found to express testisin mRNA (Fig 2A), although the levels are low when compared to the HeLa cancer line (S2C Fig). Similar levels of testisin protein were also detected in each of these endothelial cell types by immunoblotting (Fig 2B). Staining for testisin protein expression in ovary tissue samples using these and other commercial antibodies was not successful, likely due to the low levels in endothelial cells and possible cross-reactivity of the antibody with a non-specific protein in tissues (S2A Fig).

Testisin function was investigated *in vitro* using HMEC-1 cells, a cell line which retains the morphologic, phenotypic, and functional characteristics of normal human microvascular endothelial cells, such as the expression of von Willebrand Factor (vWF), CD31, ICAM-1, acetylated LDL uptake and angiogenic tubule-like formation on Matrigel, but allows greatly extended culture times without senescence while retaining endothelial characteristics [36]. Testisin was silenced in HMEC-1 cells using two independent siRNAs shown to knockdown testisin expression (S2B & S3 Figs) and compared with a negative control siRNA. Silencing of testisin significantly inhibited tube-like formation on Matrigel as observed by visual inspection after 15 hours (Fig 2C, *left*). Quantitation of tubular formations in images captured over time using NIH ImageJ software showed a persistent reduction in the total tube length throughout the time course (Fig 2C, *right*, *top*). RNA analysis of testisin expression at the completion of the assay confirmed testisin expression remained suppressed throughout the time course (Fig 2C, *right*, *bottom*). Analysis of total tube length from multiple independent experiments showed a significant (~18-fold) decrease in tube length in testisin deficient cells at the 15-hour endpoint (Fig 2D). The impaired tubule formation was not a result of a significant decrease in viability, as determined by PrestoBlue assay (Fig 2E). Image analysis of other parameters of the tube formation assay, such as the number of tubes or branches formed, all demonstrated reductions consistent with the reduction in total tube length (data not shown). Analysis of videos compiled from the time course imaging of the cultures revealed that while the control cells migrated towards each other and coalesced to form an organized capillary-like network consistent with tubule formation, testisin siRNA knockdown endothelial cells displayed random movement and failed to coalesce (see S1 and S2 Videos). Since endothelial cells have previously been shown not to proliferate on Matrigel [37] and reduced viability was not a factor, these data identify a direct role for testisin in regulating processes required for the formation of complex capillary networks.

### Vascularization of the corpus luteum occurs similarly in *Prss21*^*+/+*^ and *Prss21*^*-/-*^ mice

**Fig 3:**
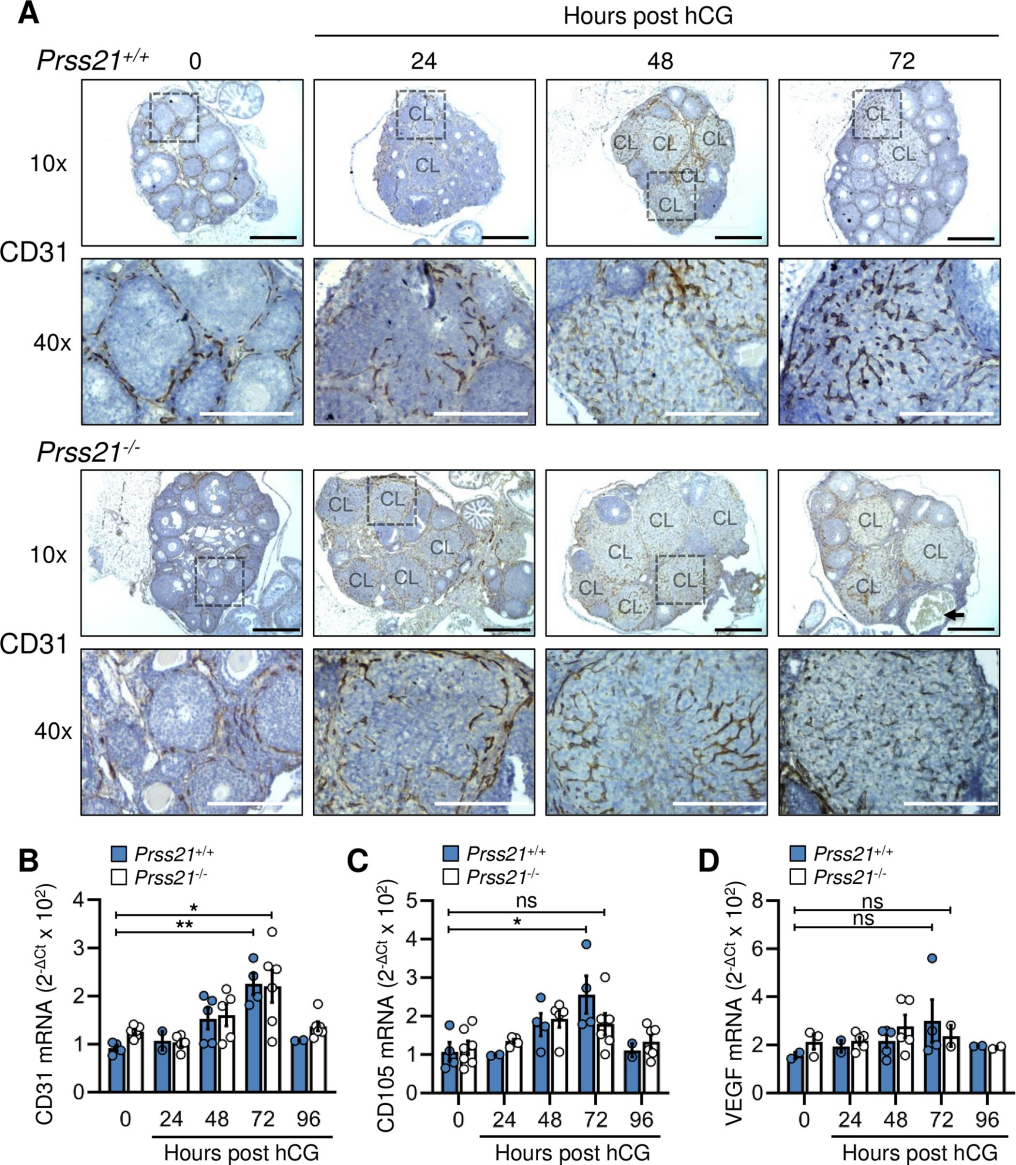
Vascularization of the corpus luteum is similar between *Prss21*^*+/+*^ and *Prss21*^*-/-*^ mice. The expression of endothelial markers in *Prss21*^*-/-*^ ovaries is not significantly different than *Prss21*^*+/+*^ ovaries over the course of luteal angiogenesis. **A)** Representative photographs of histological sections of *Prss21*^*+/+*^ and *Prss21*^*-/-*^ ovaries stained with CD31 (brown) and counterstained with hematoxylin showing widespread endothelial cell infiltration over time that is similar for both genotypes (n = 5–6 ovaries per genotype). 10x magnification, Scale bar = 500 μm; 40x magnification, Scale bar = 100 μm. Corpora lutea are labeled CL and 40x enlargement area is highlighted by the dashed box in 10x images. Arrow denotes a corpus luteum filled with blood in a *Prss21*^*-/-*^ ovary. qPCR analysis of **B)** CD31, **C)** CD105 and **D)** VEGF mRNA expression in whole ovaries during the time course of vascularization of the corpus luteum. (0h n = 4–7, 24h n = 2–5, 48h n = 4–5, 72h n = 4–6, 96h n = 2–6, where n = number of ovaries per genotype). Mean ± SEM. ***: p<0.001, **: p<0.01, *: p<0.05, ns: not significant.

Given the impairment in angiogenic tube-like formation following siRNA mediated depletion of testisin *in vitro*, the vasculature of *Prss21*^*+/+*^ and *Prss21*^*-/-*^ ovaries after gonadotropin-induced ovulation was examined for possible angiogenic abnormalities or deficiencies. Endothelial cell recruitment and neovascularization of the corpus luteum in response to hormonal treatment was investigated by immunohistological staining of *Prss21*^*+/+*^ and *Prss21*^*-/-*^ ovaries for the expression of the endothelial cell marker CD31/PECAM-1 at 0 and post hCG injection 24, 48 and 72 hours. Although within each ovary, corpus luteal formation and neovascularization is not fully synchronous, the presence of CD31 positive endothelial networks (brown staining) penetrating corpus lutea over the course of 24 to 72 hours was not ostensibly different between the 2 groups (Fig 3A). Quantitation of CD31 mRNA in total ovary homogenates as a further indication of endothelial cell content in each ovary showed no significant differences between *Prss21*^*+/+*^ and *Prss21*^*-/-*^ ovaries over the time course post-hCG (Fig 3B), indicating that endothelial cell infiltration into corpus lutea still occurs in *Prss21*^*-/-*^ ovaries. mRNA expression of the neovascular marker CD105/ENG and the vascular endothelial growth factor VEGFA were also not significantly different between *Prss21*^*+/+*^ and *Prss21*^*-/-*^ ovaries (Fig 3C & 3D). These data indicate that the overall extent of endothelial cell content and neovascularization of corpus lutea are not significantly affected by testisin deficiency. Interestingly, the mRNA expression patterns of CD31 and CD105 strongly correlated with testisin mRNA expression in the ovaries over the time course post-hCG (Figs 3B–3D vs Fig 1E), indicating an association between testisin expression and these angiogenic markers.

### Testisin depletion increases vascular permeability to FITC-albumin *in vitro*

**Fig 4:**
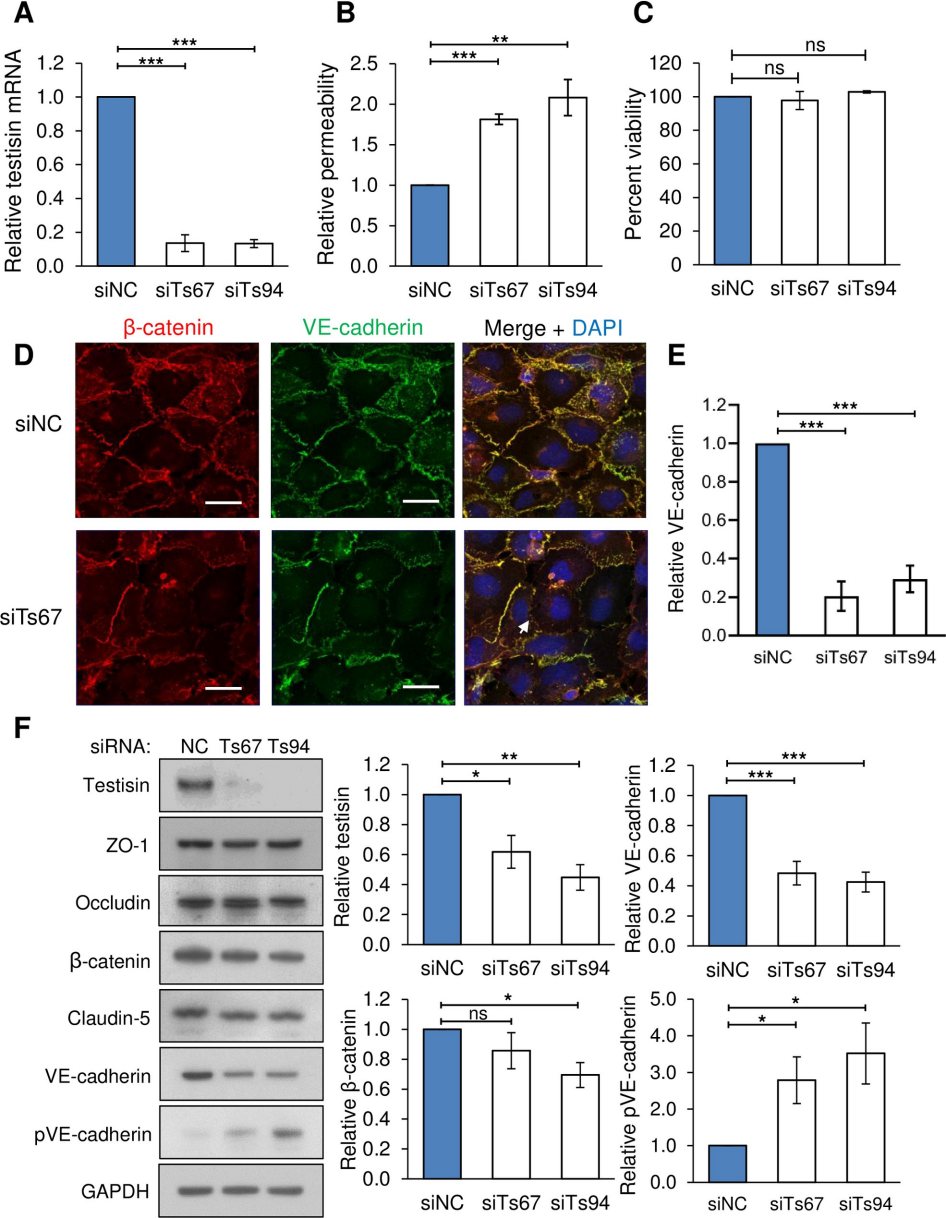
Testisin is required for barrier function in HMEC-1 endothelial monolayers. **A)** Knockdown of testisin mRNA expression by siRNA silencing in HMEC-1 monolayers, assessed by qPCR analysis. Data is represented as mean ± SEM from 3 independent experiments. **B)** Assay of permeability of testisin silenced HMEC-1 monolayers compared to control cultures, 1 hour after the addition of FITC-albumin. Data is represented as mean ± SEM from 2–3 independent experiments. **C)** Cell viability of testisin silenced HMEC-1 monolayers compared to control cultures measured at the end of the assay by PrestoBlue assay. Data is represented as mean ± SEM from 3 independent experiments. **D)** Representative confocal images of VE-cadherin and β-catenin expression in testisin siRNA silenced and control HMEC-1 monolayers grown in parallel transwell inserts along with the permeability assays, showing diminished staining and loss at cell-cell junctions (examples indicated by white arrows). Scale bar = 20 μm. **E)** Signal intensities for VE-cadherin were quantitated from EVOS images of 3 independent experiments performed in triplicate for each siRNA, using Image J. **F)** Immunoblotting and quantitation by densitometry of junctional proteins showing a significant decrease in total VE-cadherin expression and a significant increase in phospho(Tyr658)-VE cadherin in testisin silenced monolayers compared to control cultures. No significant differences in expression of the tight junction proteins ZO-1, claudin-5 and occludin were observed. Data is representative of 3–6 independent experiments. Mean ± SEM. ***: p<0.001, **: p<0.01, *: p<0.05, ns: not significant.

Noting the apparently normal recruitment and extent of endothelial cell content in the angiogenic corpus lutea, we speculated that the hemorrhagic phenotype of *Prss21*^*-/-*^ ovaries may arise from a functional vascular defect. To test this possibility, the integrity of the barrier formed by testisin siRNA silenced and negative control HMEC-1 monolayers (Fig 4A) was evaluated using the macromolecular tracer, FITC-albumin, in transwell permeability assays. One hour following the addition of FITC-albumin, the testisin silenced monolayers displayed an ~2-fold increase in permeability to FITC-albumin compared to the control monolayers (Fig 4B). This loss in barrier function was not attributable to any reduction in cell number or loss of cell viability, as measured by PrestoBlue assay (Fig 4C).

The adherens junction protein, VE-cadherin plays a key role in the control of endothelial cell barrier integrity and vascular homeostasis. Confocal microscopic examination of VE-cadherin expression in testisin siRNA silenced and control HMEC-1 monolayers showed diminished signals for VE-cadherin and loss of some intercellular junctions in the testisin silenced HMEC-1 monolayers as compared to control cultures (Fig 4D). Expression of β-catenin, which associates directly with VE-cadherin to enhance stabilization of endothelial cell barriers [38], was also diminished. Quantitation of signal intensity demonstrated a significant (3 to 5-fold) loss of VE-cadherin in testisin siRNA silenced monolayers compared with control monolayers (Fig 4E).

Analysis of protein lysates prepared from the testisin siRNA silenced and control monolayers verified that total VE-cadherin protein levels were significantly decreased in testisin silenced endothelial cell monolayers compared to the control cultures (Fig 4F). Levels of the tight junction proteins, claudin-5 and occludin, and the associated cytoplasmic protein ZO-1, did not show any significant differences between control and testisin silenced cultures (Fig 4F), suggesting that the leakiness seen with depletion of testisin is largely confined to the endothelial adherens junctions. β-catenin protein levels also appeared decreased, however to a lesser extent and reached significance with only one of the two siRNAs (Fig 4F). Immunoblotting for phospho(Tyr658)-VE-cadherin, a VE-cadherin phosphorylation site that has been associated with regulation of endothelial permeability and which mediates VE-cadherin internalization [39], was significantly increased in testisin silenced endothelial cell monolayers compared to the control cells, suggesting that the observed loss of total VE-cadherin may be mediated by receptor phosphorylation, internalization and degradation [40].

### Testisin loss is associated with increased Evans blue extravasation and decreased intercellular VE-cadherin during luteal angiogenesis

**Fig 5:**
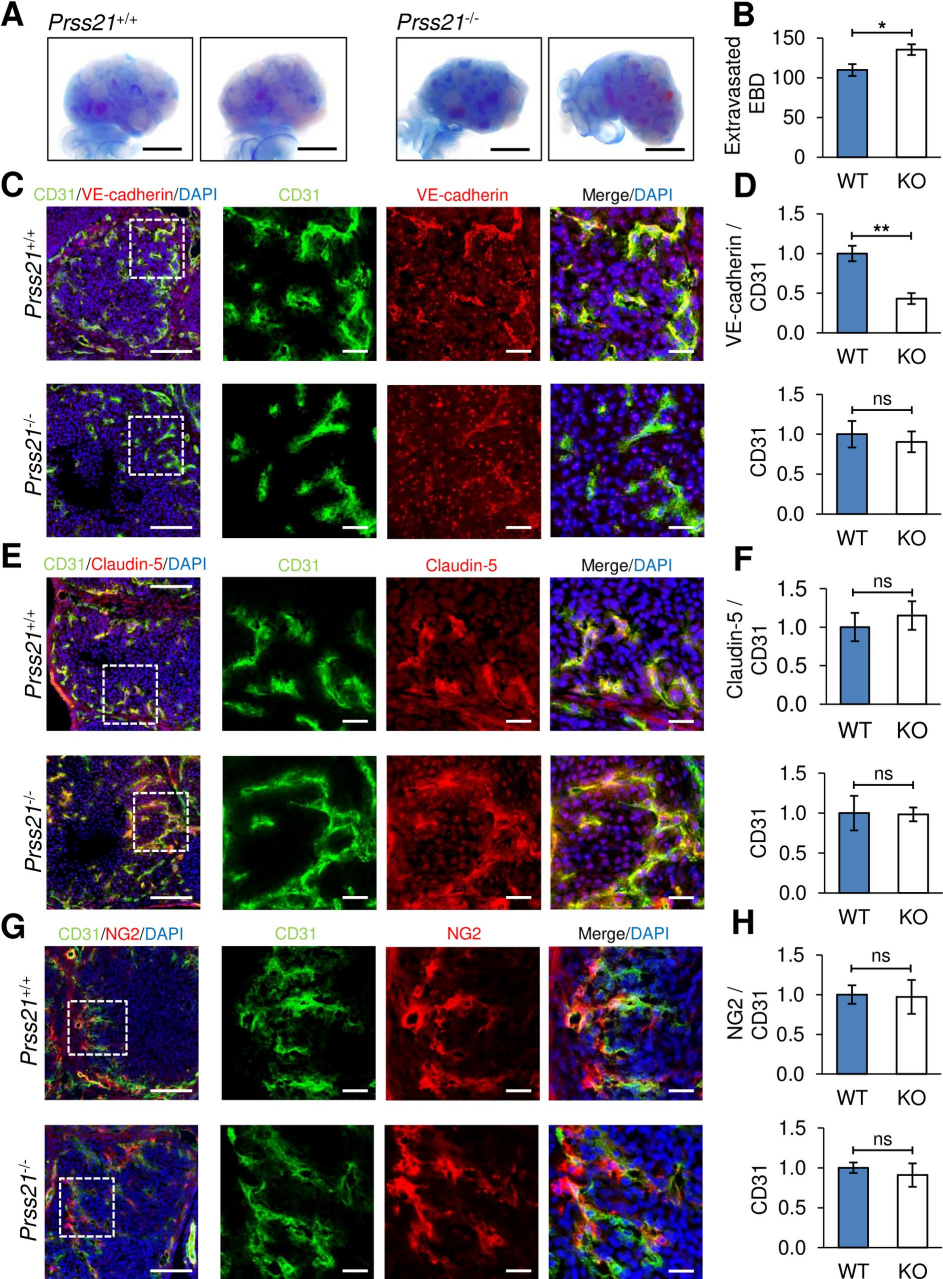
*Prss21*^*-/-*^ mice display increased vascular permeability during luteal angiogenesis and decreased endothelial VE-cadherin expression. **A)** Representative images of extravasated Evans blue dye in whole mount *Prss21*^*+/+*^ and *Prss21*^*-/-*^ ovaries, 24 hours post hCG. *Prss21*^*-/-*^ ovaries exhibited an increased number and size of blue-stained extravascular areas and less dye negative areas following PBS perfusion. Scale bar = 1000 μm. **B)** Extravasated Evans blue dye as a measure of vascular permeability at 24 hours post hCG by quantitation of dye extracted from perfused ovaries, presented as (μg of Evans blue dye)/(g of tissue), n = 8–9 mice per genotype. **C)** Representative images of VE-cadherin expression in *Prss21*^*+/+*^ and *Prss21*^*-/-*^ ovaries, 24 hours post hCG. Frozen sections were prepared and immunostained for endothelial cells (CD31, green), VE-cadherin (red), and nuclei (DAPI). Left panel shows images of corpora lutea (scale bar = 100 μm), followed by a 5X enlargement of the area highlighted by the dashed white box (scale bar = 20 μm). **D)** Analysis of fluorescence intensity values from least 3 corpora lutea per ovary, from at least 3 ovaries per genotype, revealed similar levels of CD31 and significantly lower VE-cadherin expression in *Prss21*^*-/-*^ ovaries, compared with *Prss21*^*+/+*^ ovaries. **E)** Claudin-5 staining (red) appeared similar between *Prss21*^*+/+*^ and *Prss21*^*-/-*^ ovaries, and **F)** fluorescence intensity quantitation was not significantly different between genotypes. **G)** Pericyte staining (NG2, red) and endothelial association appeared similar between *Prss21*^*+/+*^ and *Prss21*^*-/-*^ ovaries, and **H)** fluorescence intensity quantitation was not significantly different between genotypes. Mean ± SEM. ***: p<0.001, **: p<0.01, *: p<0.05. ns, not significant.

The barrier integrity of the luteal neovasculature was investigated by assay of Evans blue dye extravasation. Hormonally treated mice at 24 hours post-hCG were injected retro-orbitally with Evans blue dye, and after 30 min, perfused with PBS, prior to removal of the ovaries. Visual inspection revealed that *Prss21*^*-/-*^ ovaries appeared more permeable to the dye, as indicated by the increased number, size, and intensity of Evans blue positive areas as compared to *Prss21*^*+/+*^ ovaries (Fig 5A). Quantitation of Evans blue dye accumulation following TCA extraction of whole ovaries demonstrated a significant increase in tissue deposition of extravasated dye in *Prss21*^*-/-*^ ovaries as compared to *Prss21*^*+/+*^ controls (Fig 5B).

Examination of VE-cadherin at the inter-endothelial junctions in the neovasculature of corpora lutea at 24 hours post-hCG by co-immunofluorescent staining for VE-cadherin and CD31 revealed appreciably less intense VE-cadherin staining within the newly forming neovasculature of corpora lutea of *Prss21*^*-/-*^ ovaries as compared to *Prss21*^*+/+*^ controls (Fig 5C). Quantitation of the fluorescent intensity of VE-cadherin staining that was clearly associated with CD31 positive endothelial cells, was reduced by ~2.3-fold in *Prss21*^*-/-*^ ovaries (Fig 5D). VE-cadherin staining in the mature vasculature surrounding the multiple corpora lutea appeared normal in *Prss21*^*-/-*^ ovaries as compared to *Prss21*^*+/+*^ ovaries (S4 Fig), indicating that expression of VE-cadherin is reduced specifically within the corpora lutea during microvascular angiogenesis and is not globally suppressed. CD31 fluorescence staining in the corpora lutea did not vary significantly between *Prss21*^*+/+*^ and *Prss21*^*-/-*^ ovaries, in agreement with the immunohistological findings (Fig 3A). Similarly, the fluorescence intensity of the tight junction protein claudin-5 detected at the inter-endothelial junctions was similar between *Prss21*^*+/+*^ and *Prss21*^*-/-*^ ovaries (Fig 5E and 5F) which is consistent the absence of changes in claudin-5 in the testisin silenced HMEC-1 monolayers (Fig 4F). While reduced pericyte recruitment to the neovasculature can contribute to increased vascular permeability *in vivo* [41], we detected no differences in the staining and quantitation of the pericyte marker NG2 between the two genotypes (Fig 5G and 5H).

Together these data demonstrate a functional role for testisin in the regulation of neovascular integrity and permeability through modulation of intercellular VE-cadherin, that likely underlies the hemorrhagic phenotype associated with *Prss21*^*-/-*^ ovaries during luteal angiogenesis.

## Discussion

Angiogenesis and vascular remodeling are dynamic processes involving coordinated endothelial cell behavior that is tightly controlled at many levels. Here, we show that testisin, a membrane anchored serine protease expressed in microvascular endothelial cells, is a novel modulator of microvascular permeability and angiogenesis, and plays a critical role in preventing early vascular leak during the early rapid vascularization of the corpus luteum. Depletion of testisin impairs endothelial cell angiogenic tubule-like formation and reorganization on Matrigel and enhances endothelial cell barrier permeability *in vitro*. Together these data suggest that testisin plays a key role in regulating the formation and functional integrity of newly forming capillaries during physiological angiogenesis.

Following ovulation, endothelial cells rapidly invade the corpus luteum to develop a network of angiogenic neovasculature that promotes the production of high amounts of progesterone. We found that a typical defect in *Prss21*^-/-^ ovaries was the sustained occurrence of multiple large hemorrhages. This could not be explained by changes in endothelial cell composition or endothelial cell morphologies but was associated with a functional defect in vascular integrity, as evidenced by increased vascular permeability to macromolecular albumin tracers, investigated both *in vivo* and *in vitro*. An increase in vessel fragility can cause hemorrhage when newly forming vessels are subjected to shear stress [38]. In mice lacking testisin the observed decrease in VE-cadherin expression during angiogenesis could explain this increased fragility. In fact, it has previously been found that the administration of anti-VE-cadherin antibodies in adult mice leads to a dramatic increase in vascular permeability, vascular fragility and hemorrhages [42]. It is possible that in testisin deficient mice during early angiogenesis when developing small capillaries are exposed to hemodynamic shear stress, weakened inter-endothelial junctions may break causing vascular leakage. This could explain the localized and persistent occurrence of vascular hemorrhages in the developing corpus luteum of *Prss21*^*-/-*^ mice. As the process of vascularization progresses, recruited pericytes could serve to reinforce the vascular structure and enable capillary stabilization [43].

Cell-cell adhesion is critically important for maintaining vascular integrity and preventing leakage of blood, antigenic materials and cells into tissues. Vascular permeability through the paracellular pathway is largely controlled by intercellular junctions composed of transmembrane proteins that include claudin-5 and occludin, the cytoplasmic zonula occludens (ZO-1) and the adherens protein, VE-cadherin. Of these junctional components, only VE-cadherin was persistently decreased in the testisin siRNA silenced cultures. VE-cadherin clustering at cell-cell contacts controls vascular permeability and is also a transducer of intracellular signals critical to maintaining proper endothelial cell function [44]. Reduced junctional VE-cadherin is associated with reduced cell-cell adhesion and increased paracellular permeability [45] and antibody-mediated blockade of VE-cadherin has been shown to inhibit angiogenic capillary tube formation in fibrin and collagen gels [46]. Tyrosine phosphorylation of the VE-cadherin cytoplasmic domain is observed in weak endothelial barriers and is also induced in tight barriers after exposure to endothelial barrier disrupting agents such as VEGF, cytokines and leukocyte proteases [11, 45]. Multiple specific tyrosine residues of VE-cadherin are phosphorylated depending on the stimulus and are thought to cause the disruption of interactions with adherens junction molecules such as β- and p120-catenins, proteins that are known to stabilize VE-cadherin localization at cell junctions. At the same time, serine phosphorylation of VE-cadherin is thought to promote its association with β-arrestin-2, inducing its internalization and turnover [47]. The signaling pathways that lead to VE-cadherin phosphorylation at particular residues are not well understood, however, phosphorylation of VE-cadherin on Tyr 658, which was observed following testisin siRNA knockdown, is thought to be mediated by Src family kinases [48, 49]. The signaling pathways activated by testisin expression in endothelial cells that could suppress the activity of Src tyrosine kinases are currently unknown. Whether testisin alters the phosphorylation state of other sites in VE-cadherin also remains to be investigated.

Testisin is a serine protease that anchors on the endothelial cell surface, where its substrates are mostly unknown. It has specificity for cleavage after basic amino acids with an apparent preference for arginine and is capable of activating the G-protein coupled protease activated receptor type-2 (PAR-2) [50, 51]. Whether testisin alters PAR-2 signaling pathways to regulate VE-cadherin expression remains to be investigated. Studies that have linked PAR-2 activation, altered VE-cadherin expression, and permeability changes [52–54] show that PAR-2 activation causes the loss of junctional VE-cadherin resulting in barrier disruption, in contrast to the observed barrier protective activity of testisin observed in the present study. Future studies are required to investigate whether PAR-2 regulation could be involved in testisin-mediated barrier protection of the microvasculature, or whether other substrates are involved.

Endothelial function and angiogenesis are important for adult homeostasis. Physiological angiogenesis occurs in the adult during the ovarian cycle and in physiological repair processes such as wound healing. Endothelial dysfunction is associated with several pathophysiological conditions, including atherosclerosis, hypertension and diabetes. How endothelial cells proliferate and arrange suitably to shape functional vascular networks is still poorly understood. Testisin represents a novel component of an angiogenic pathway that is essential for vascular integrity during physiological angiogenesis. A better understanding of the role of testisin in angiogenic processes will be important since testisin-based therapeutic strategies could have application for preventing, treating or altering the progression of vascular diseases.

## Supporting information

S1 Fig Generation of *Prss21* knockout mice. Three targeted knockout KOMP ES cell lines, Prss21^tm1a(KOMP)Wtsi^ (Project ID CSD47003; RRID:MMRRC_060817-UCD) were obtained from the Mutant Mouse Resource and Research Center (MMRRC) (https://www.mmrrc.org/catalog/cellLineSDS.php?mmrrc_id=60817). Two germ line testisin-deficient chimeras were generated (A10 and G8) and were mated with wild-type C57BL/6 mice to generate heterozygous carriers of the targeted allele which were then mated to produce wild-type (*Prss21*^*+/+*^), heterozygous (*Prss21*^*+/-*^), and testisin knockout (*Prss21*^*-/-*^) mice. Colonies G10 and A10 were bred and maintained as two separate colonies. Breeding pairs of het x het crosses were used to generate *Prss21*^*+/+*^ and *Prss21*^*-/-*^ mice for experiments. *Prss21*^*−/−*^, *Prss21*^*−/+*^ and *Prss21*^*−/−*^ F(2) progeny were born at the expected Mendelian ratio of 1:2:1 (data not shown). The *Prss21*^*−/−*^ mice appear to develop normally and have no identifiable behavior abnormalities or obvious adverse phenotype as has been observed previously^21^. A) PCR genotyping of littermates from a het x het breeding pair. Genotyping of mice was performed by DNA isolation from tail clippings of mice using the RedExtract-n-amp kit (Sigma-Aldrich), and PCR amplification with the genotyping primers: mTestisin forward (F4): AAC CTT GCT CAA CCG CCG C; mTestisin WT reverse (R3): TGG GGC TCA GGA AAA TAT CT; mTestisin KO reverse (LAR3): CAC AAC GGG TTC TTC TGT TAG TTC. B) PCR amplification of RNA isolated and reverse-transcribed from the testes of *Prss21*^*+/+*^ and *Prss21*^*-/-*^ mice demonstrating targeted disruption of testisin transcription. The cDNA was amplified with primers F4 and R3.
(PDF)

S2 Fig Analysis of relative testisin expression in cell lines and determination of the specificity of the anti-testisin monoclonal antibody, D9.1. A) A hybridoma cell line expressing the monoclonal anti-testisin antibody D9.1 was purchased from the ATCC (Pro104.D9.1; ATCC, Manassas, VA). The cell line was cultured and the antibody purified from conditioned media using Protein G-Sepharose by standard methods. Depicted is an immunoblot analysis of lysates prepared from testes of *Prss21*^*+/+*^ (WT) and *Prss21*^*-/-*^ (KO) male mice probed with purified anti-testisin D9.1 antibody and reprobed with β-actin as a control for loading. The antibody detects a non-specific protein in the tissue lysates. The data is representative of two independent experiments. B) Immunoblot analysis of cell lysates prepared from HeLa cells transfected with control siRNA (siNC), or two testisin targeted siRNAs (siTs67 and siTs94). Blots were probed with purified anti-testisin D9.1 antibody. Samples were rerun and probed for β-actin. The data is representative of 3 independent experiments. C) qPCR analysis of testisin mRNA expression in HMEC-1 cells compared to ES-2 and HeLa tumor cell lines. HeLa cells express relatively high levels of testisin while ES-2 cells express negligible amounts.
(PDF)

S3 Fig Evaluation of testisin knockdown by three testisin-targeted siRNAs in HMEC-1 cells. A) qPCR analysis of testisin mRNA relative to siNC after normalizing to GAPDH at 48 hours post-transfection after knockdown with 5nM of siTs67, siTs68, siTs94 and the non-targeted siNC control. Results are from technical replicates and are representative of two independent experiments. B) Cell viability after siRNA knockdown measured using PrestoBlue 72hrs post-transfection. Signals were normalized to the siRNA NC cells and are representative of two independent experiments. C) Immunoblot analysis of testisin and control GAPDH protein expression in HMEC-1 cells after silencing with the three testisin-targeted siRNAs at 72 hours post transfection. Graph shows densitometric analysis of testisin normalized to GAPDH and relative to siNC. The siRNAs, siTs67, siTs94 effectively silenced testisin expression without loss of viability, and were selected for use subsequent experiments. qPCR and viability graphs show mean ± SD. Densitometry graphs show mean ± SEM from 2 independent experiments. * p<0.05 ** p<0.01, unpaired *t*-test.
(PDF)

S4 Fig VE-cadherin staining of non-angiogenic mature vasculature in *Prss21*^*+/+*^ and *Prss21*^*+/+*^ ovaries is similar. A) Frozen sections (7μm) from OCT blocks of *Prss21*^+/+^ and *Prss21*^-/-^ ovaries recovered at 24 hours post hCG were prepared and stained for CD31, VE-cadherin, and nuclei (DAPI). Analysis of several sections, from at least 3 ovaries, revealed similar intensity and staining patterns for VE-cadherin in the larger existing vasculature. Images were taken at 20x using an EVOS FL2 (ThermoFisher). Scale bars = 100 μm.
(PDF)

S1 Video Time course imaging of EC tubule formation assay: siNC vs siTs67. (AVI)

S2 Video Time course imaging of EC tubule formation assay: siNC vs siTs94. (AVI)

S1 Raw Images Original Images for blots and gels. (PDF)
