# Supplementary material for: Testisin/Prss21 deficiency causes increased vascular permeability and a hemorrhagic phenotype during luteal angiogenesis
Source: PLoS One. 2020 Jun 8;15(6):e0234407. doi: 10.1371/journal.pone.0234407 (PMC7279603; doi:10.1371/journal.pone.0234407)
Supplement: S1 Fig — Three targeted knockout KOMP ES cell lines, Prss21tm1a(KOMP)Wtsi (Project ID CSD47003; RRID:MMRRC_060817-UCD) were obtained from the Mutant Mouse Resource and Research Center (MMRRC) (https://www.mmrrc.org/catalog/cellLineSDS.php?mmrrc_id=60817). Two germ line testisin-deficient chimeras were generated (A10 and G8) and were mated with wild-type C57BL/6 mice to generate heterozygous carriers of the targeted allele which were then mated to produce wild-type (Prss21+/+), heterozygous (Prss21+/-), and testisin knockout (Prss21-/-) mice. Colonies G10 and A10 were bred and maintained as two separate colonies. Breeding pairs of het x het crosses were used to generate Prss21+/+ and Prss21-/- mice for experiments. Prss21−/−, Prss21−/+ and Prss21−/− F(2) progeny were born at the expected Mendelian ratio of 1:2:1 (data not shown). The Prss21−/− mice appear to develop normally and have no identifiable behavior abnormalities or obvious adverse phenotype as has been observed previously21. A) PCR genotyping of littermates from a het x het breeding pair. Genotyping of mice was performed by DNA isolation from tail clippings of mice using the RedExtract-n-amp kit (Sigma-Aldrich), and PCR amplification with the genotyping primers: mTestisin forward (F4): AAC CTT GCT CAA CCG CCG C; mTestisin WT reverse (R3): TGG GGC TCA GGA AAA TAT CT; mTestisin KO reverse (LAR3): CAC AAC GGG TTC TTC TGT TAG TTC. B) PCR amplification of RNA isolated and reverse-transcribed from the testes of Prss21+/+ and Prss21-/- mice demonstrating targeted disruption of testisin transcription. The cDNA was amplified with primers F4 and R3. (PDF) [file pone.0234407.s001.pdf]

Supplementary Figure S1

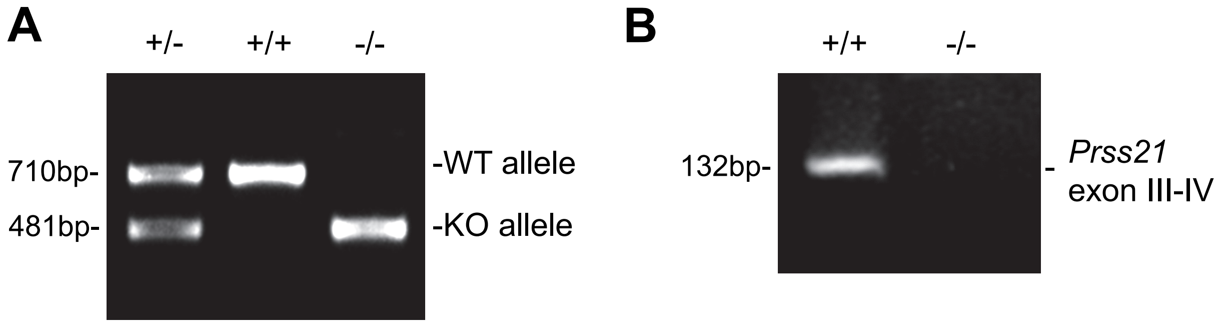

Supplementary Figure S1. Generation of *Prss21* knockout mice. Three targeted knockout KOMP ES cell lines, Prss21$^{tm1a(KOMP)Wtsi}$ (Project ID CSD47003; RRID:MMRRC_060817-UCD) were obtained from the Mutant Mouse Resource and Research Center (MMRRC) (https://www.mmrrc.org/catalog/cellLineSDS.php?mmrrc_id=60817). Two germ line testisin-deficient chimeras were generated (A10 and G8) and were mated with wild-type C57BL/6 mice to generate heterozygous carriers of the targeted allele which were then mated to produce wild-type (*Prss21*$^{+/+}$), heterozygous (*Prss21*$^{+/-}$), and testisin knockout (*Prss21*$^{-/-}$) mice. Colonies G10 and A10 were bred and maintained as two separate colonies. Breeding pairs of het x het crosses were used to generate *Prss21*$^{+/+}$ and *Prss21*$^{-/-}$ mice for experiments. *Prss21*$^{-/-}$, *Prss21*$^{-/+}$ and *Prss21*$^{-/-}$ F(2) progeny were born at the expected Mendelian ratio of 1:2:1 (data not shown). The *Prss21*$^{-/-}$ mice appear to develop normally and have no identifiable behavior abnormalities or obvious adverse phenotype as has been observed previously[21]. **A)** PCR genotyping of littermates from a het x het breeding pair. Genotyping of mice was performed by DNA isolation from tail clippings of mice using the RedExtract-n-amp kit (Sigma-Aldrich), and PCR amplification with the genotyping primers: mTestisin forward (F4): AAC CTT GCT CAA CCG CCG C; mTestisin WT reverse (R3): TGG GGC TCA GGA AAA TAT CT; mTestisin KO reverse (LAR3): CAC AAC GGG TTC TTC TGT TAG TTC. **B)** PCR amplification of RNA isolated and reverse-transcribed from the testes of *Prss21*$^{+/+}$ and *Prss21*$^{-/-}$ mice demonstrating targeted disruption of testisin transcription. The cDNA was amplified with primers F4 and R3.
