# Supplementary material for: Testisin/Prss21 deficiency causes increased vascular permeability and a hemorrhagic phenotype during luteal angiogenesis
Source: PLoS One. 2020 Jun 8;15(6):e0234407. doi: 10.1371/journal.pone.0234407 (PMC7279603; doi:10.1371/journal.pone.0234407)
Supplement: S2 Fig — A) A hybridoma cell line expressing the monoclonal anti-testisin antibody D9.1 was purchased from the ATCC (Pro104.D9.1; ATCC, Manassas, VA). The cell line was cultured and the antibody purified from conditioned media using Protein G-Sepharose by standard methods. Depicted is an immunoblot analysis of lysates prepared from testes of Prss21+/+ (WT) and Prss21-/- (KO) male mice probed with purified anti-testisin D9.1 antibody and reprobed with β-actin as a control for loading. The antibody detects a non-specific protein in the tissue lysates. The data is representative of two independent experiments. B) Immunoblot analysis of cell lysates prepared from HeLa cells transfected with control siRNA (siNC), or two testisin targeted siRNAs (siTs67 and siTs94). Blots were probed with purified anti-testisin D9.1 antibody. Samples were rerun and probed for β-actin. The data is representative of 3 independent experiments. C) qPCR analysis of testisin mRNA expression in HMEC-1 cells compared to ES-2 and HeLa tumor cell lines. HeLa cells express relatively high levels of testisin while ES-2 cells express negligible amounts. (PDF) [file pone.0234407.s002.pdf]

Supplementary Figure S2

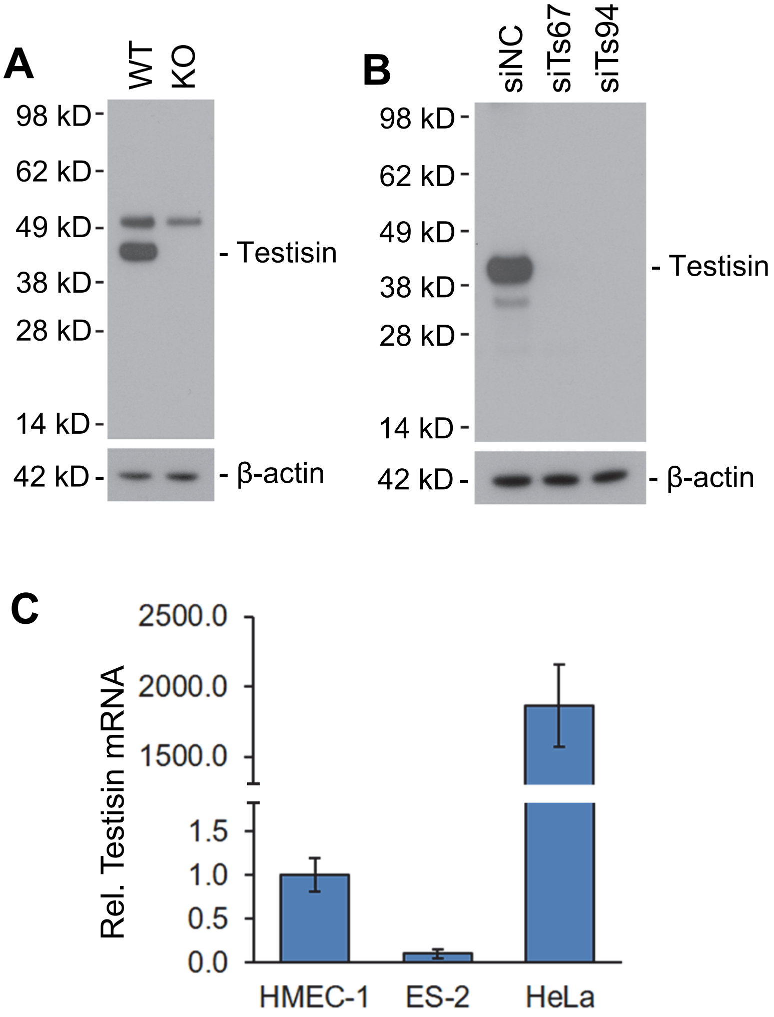

Supplementary Figure S2. Analysis of relative testisin expression in cell lines and determination of the specificity of the anti-testisin monoclonal antibody, D9.1. A) A hybridoma cell line expressing the monoclonal anti-testisin antibody D9.1 was purchased from the ATCC (Pro104.D9.1; ATCC, Manassas, VA). The cell line was cultured and the antibody purified from conditioned media using Protein G-Sepharose by standard methods. Depicted is an immunoblot analysis of lysates prepared from testes of *Prss21*$^{+/+}$ (WT) and *Prss21*$^{-/-}$ (KO) male mice probed with purified anti-testisin D9.1 antibody and reprobed with β-actin as a control for loading. The antibody detects a non-specific protein in the tissue lysates. The data is representative of two independent experiments. **B)** Immunoblot analysis of cell lysates prepared from HeLa cells transfected with control siRNA (siNC), or two testisin targeted siRNAs (siTs67 and siTs94). Blots were probed with purified anti-testisin D9.1 antibody. Samples were rerun and probed for β-actin. The data is representative of 3 independent experiments. **C)** qPCR analysis of testisin mRNA expression in HMEC-1 cells compared to ES-2 and HeLa tumor cell lines. HeLa cells express relatively high levels of testisin while ES-2 cells express negligible amounts.
