# Supplementary material for: Testisin/Prss21 deficiency causes increased vascular permeability and a hemorrhagic phenotype during luteal angiogenesis
Source: PLoS One. 2020 Jun 8;15(6):e0234407. doi: 10.1371/journal.pone.0234407 (PMC7279603; doi:10.1371/journal.pone.0234407)
Supplement: S3 Fig — A) qPCR analysis of testisin mRNA relative to siNC after normalizing to GAPDH at 48 hours post-transfection after knockdown with 5nM of siTs67, siTs68, siTs94 and the non-targeted siNC control. Results are from technical replicates and are representative of two independent experiments. B) Cell viability after siRNA knockdown measured using PrestoBlue 72hrs post-transfection. Signals were normalized to the siRNA NC cells and are representative of two independent experiments. C) Immunoblot analysis of testisin and control GAPDH protein expression in HMEC-1 cells after silencing with the three testisin-targeted siRNAs at 72 hours post transfection. Graph shows densitometric analysis of testisin normalized to GAPDH and relative to siNC. The siRNAs, siTs67, siTs94 effectively silenced testisin expression without loss of viability, and were selected for use subsequent experiments. qPCR and viability graphs show mean ± SD. Densitometry graphs show mean ± SEM from 2 independent experiments. * p<0.05 ** p<0.01, unpaired t-test. (PDF) [file pone.0234407.s003.pdf]

Supplementary Figure S3

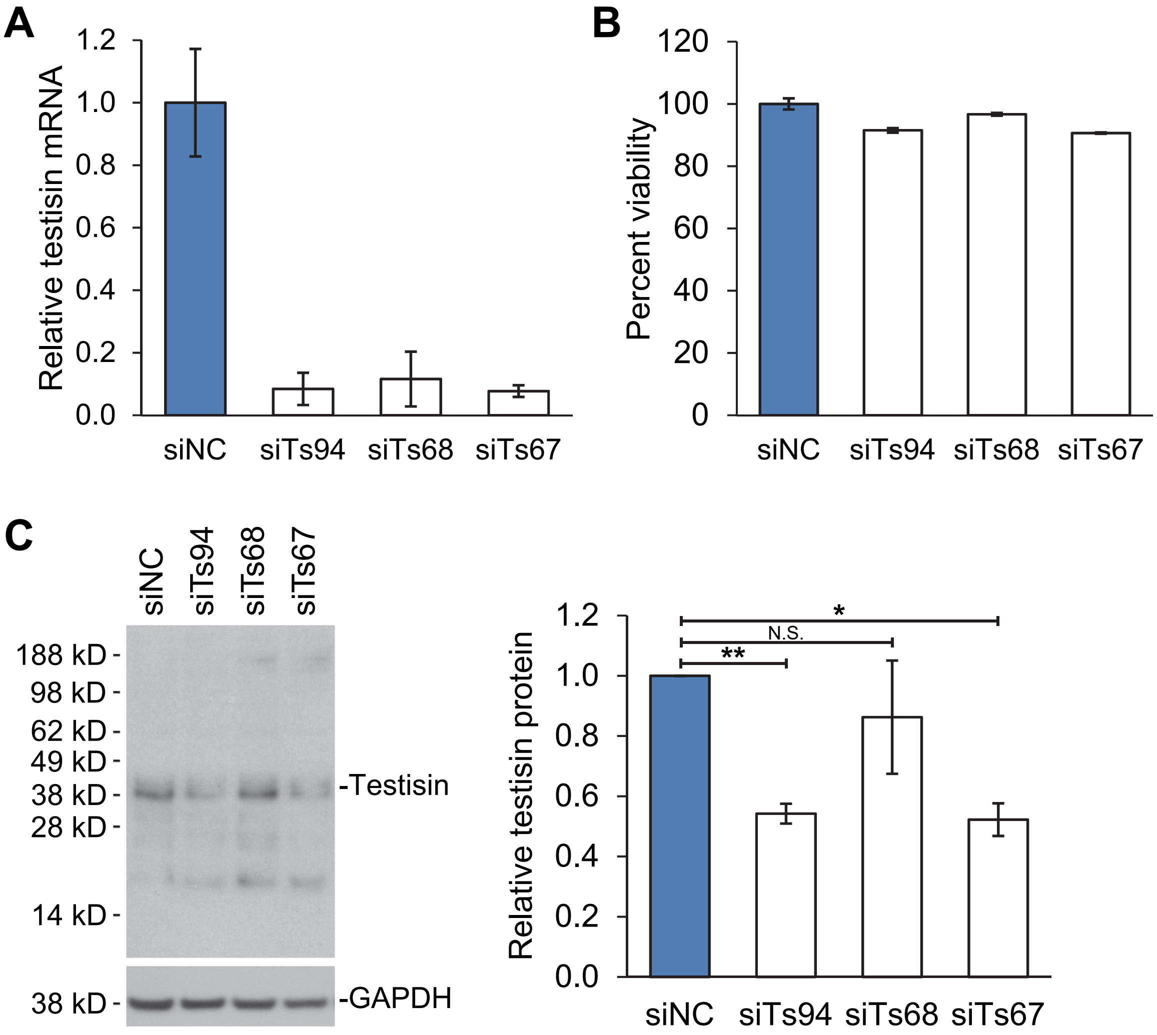

Supplementary Figure S3. Evaluation of testisin knockdown by three testisin-targeted siRNAs in HMEC-1 cells. A) qPCR analysis of testisin mRNA relative to siNC after normalizing to GAPDH at 48 hours post-transfection after knockdown with 5nM of siTs67, siTs68, siTs94 and the non-targeted siNC control. Results are from technical replicates and are representative of two independent experiments. **B)** Cell viability after siRNA knockdown measured using PrestoBlue 72hrs post-transfection. Signals were normalized to the siRNA NC cells and are representative of two independent experiments. **C)** Immunoblot analysis of testisin and control GAPDH protein expression in HMEC-1 cells after silencing with the three testisin-targeted siRNAs at 72 hours post transfection. Graph shows densitometric analysis of testisin normalized to GAPDH and relative to siNC. The siRNAs, siTs67, siTs94 effectively silenced testisin expression without loss of viability, and were selected for use subsequent experiments. qPCR and viability graphs show mean +/- SD. Densitometry graphs show mean +/- SEM from 2 independent experiments. * $p<0.05$ ** $p<0.01$, unpaired *t*-test.
