# Supplementary material for: Testisin/Prss21 deficiency causes increased vascular permeability and a hemorrhagic phenotype during luteal angiogenesis
Source: PLoS One. 2020 Jun 8;15(6):e0234407. doi: 10.1371/journal.pone.0234407 (PMC7279603; doi:10.1371/journal.pone.0234407)
Supplement: S4 Fig — A) Frozen sections (7μm) from OCT blocks of Prss21+/+ and Prss21-/- ovaries recovered at 24 hours post hCG were prepared and stained for CD31, VE-cadherin, and nuclei (DAPI). Analysis of several sections, from at least 3 ovaries, revealed similar intensity and staining patterns for VE-cadherin in the larger existing vasculature. Images were taken at 20x using an EVOS FL2 (ThermoFisher). Scale bars = 100 μm. (PDF) [file pone.0234407.s004.pdf]

Supplementary Figure S4

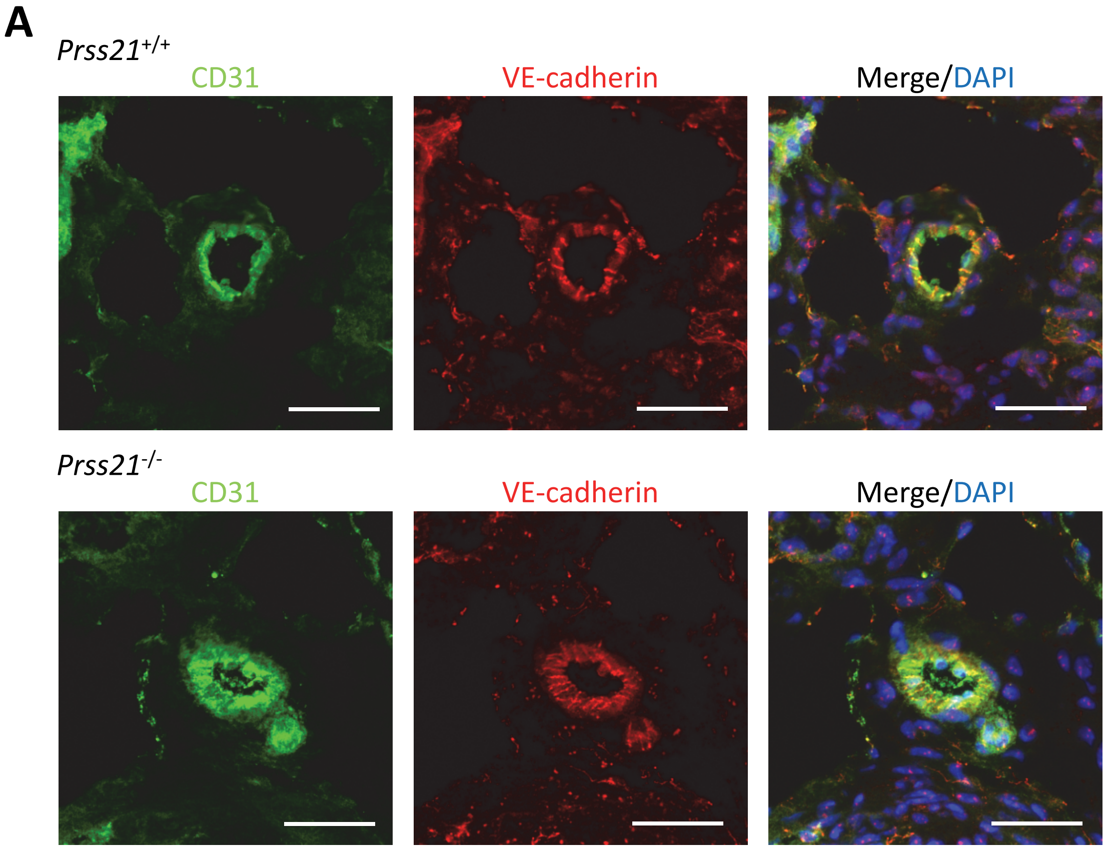

Supplementary Figure S4. VE-cadherin staining of non-angiogenic mature vasculature in *Prss21*$^{+/+}$ and *Prss21*$^{+/+}$ ovaries is similar. A) Frozen sections (7µm) from OCT blocks of *Prss21*$^{+/+}$ and *Prss21*$^{-/-}$ ovaries recovered at 24 hours post hCG were prepared and stained for CD31, VE-cadherin, and nuclei (DAPI). Analysis of several sections, from at least 3 ovaries, revealed similar intensity and staining patterns for VE-cadherin in the larger existing vasculature. Images were taken at 20x using an EVOS FL2 (ThermoFisher). Scale bars = 100 µm.
