# Supplementary material for: Testisin/Prss21 deficiency causes increased vascular permeability and a hemorrhagic phenotype during luteal angiogenesis
Source: PLoS One. 2020 Jun 8;15(6):e0234407. doi: 10.1371/journal.pone.0234407 (PMC7279603; doi:10.1371/journal.pone.0234407)
Supplement: S1 Raw Images — (PDF) [file pone.0234407.s007.pdf]

S1_raw_images

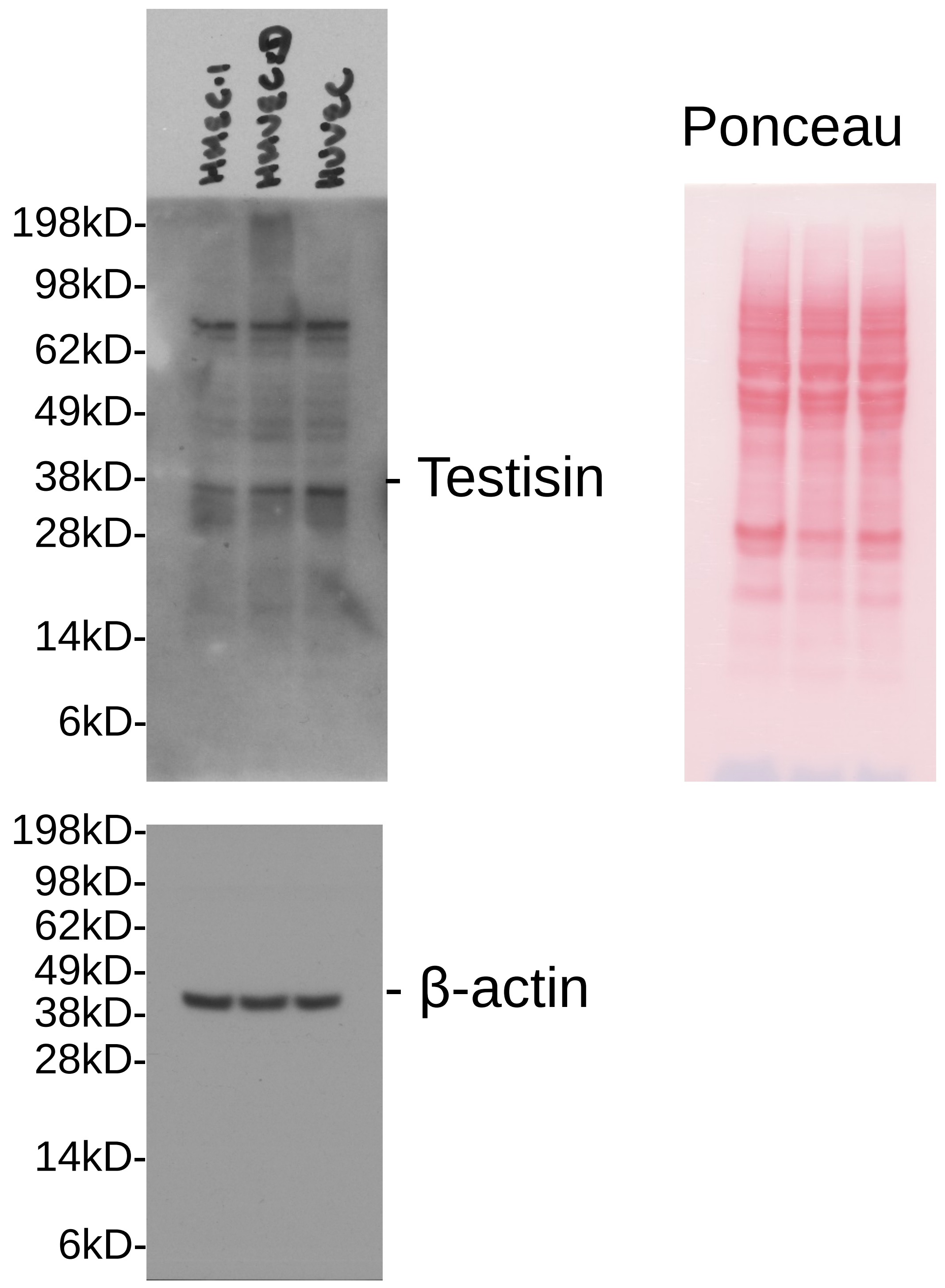

Uncropped images of immunoblots presented in Figure 2B. The Ponceau S stain is of the original testisin blot indicating total protein transferred to PVDF. Samples were rerun and a new blot probed for β-actin.

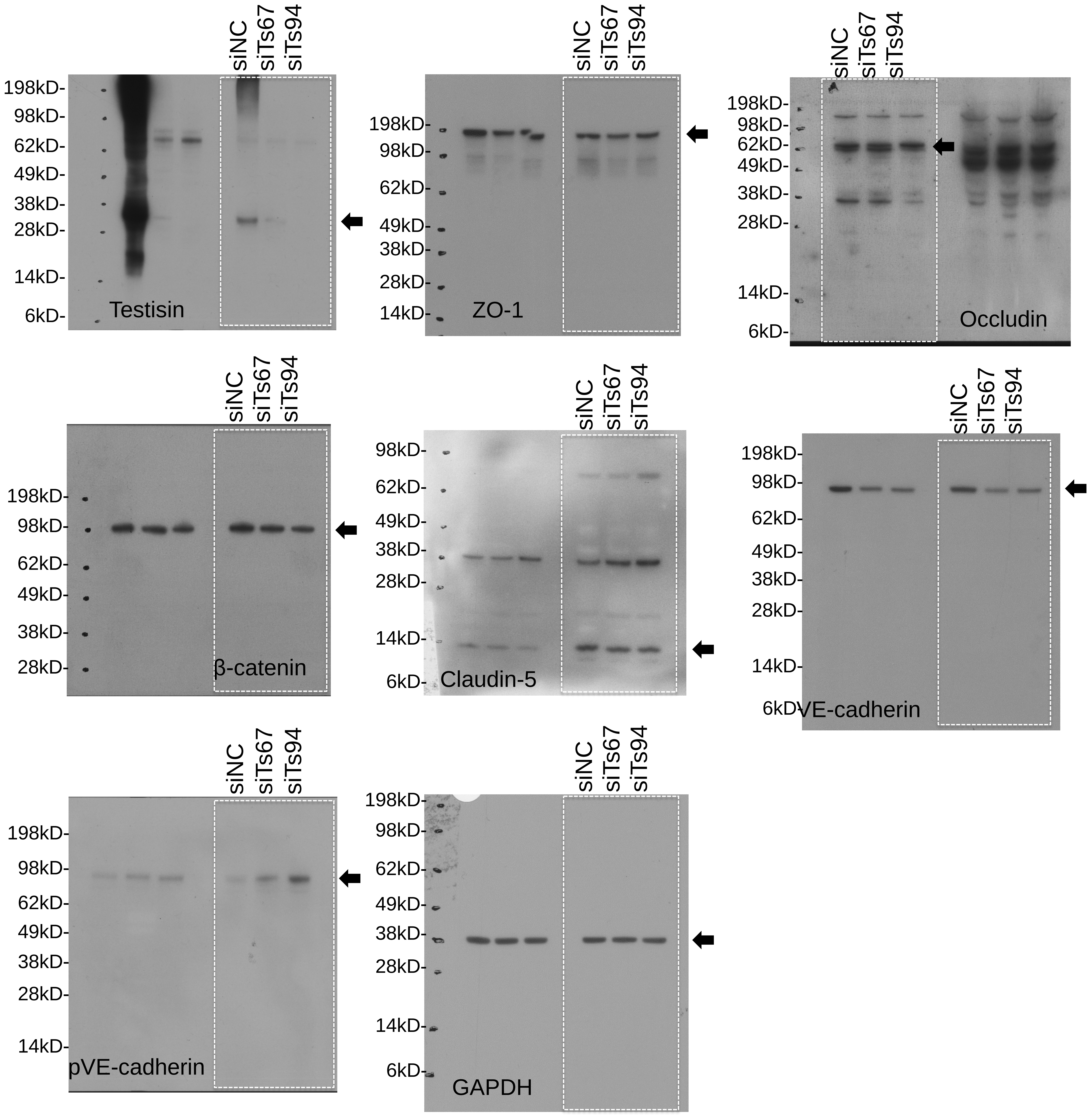

Uncropped images of immunoblots presented in Figure 4E (highlighted by dashed white boxes). Testisin, ZO-1, Occludin, β-Catenin, Claudin-5, VE-Cadherin, pVE-Cadherin, and GAPDH are indicated by black arrows.

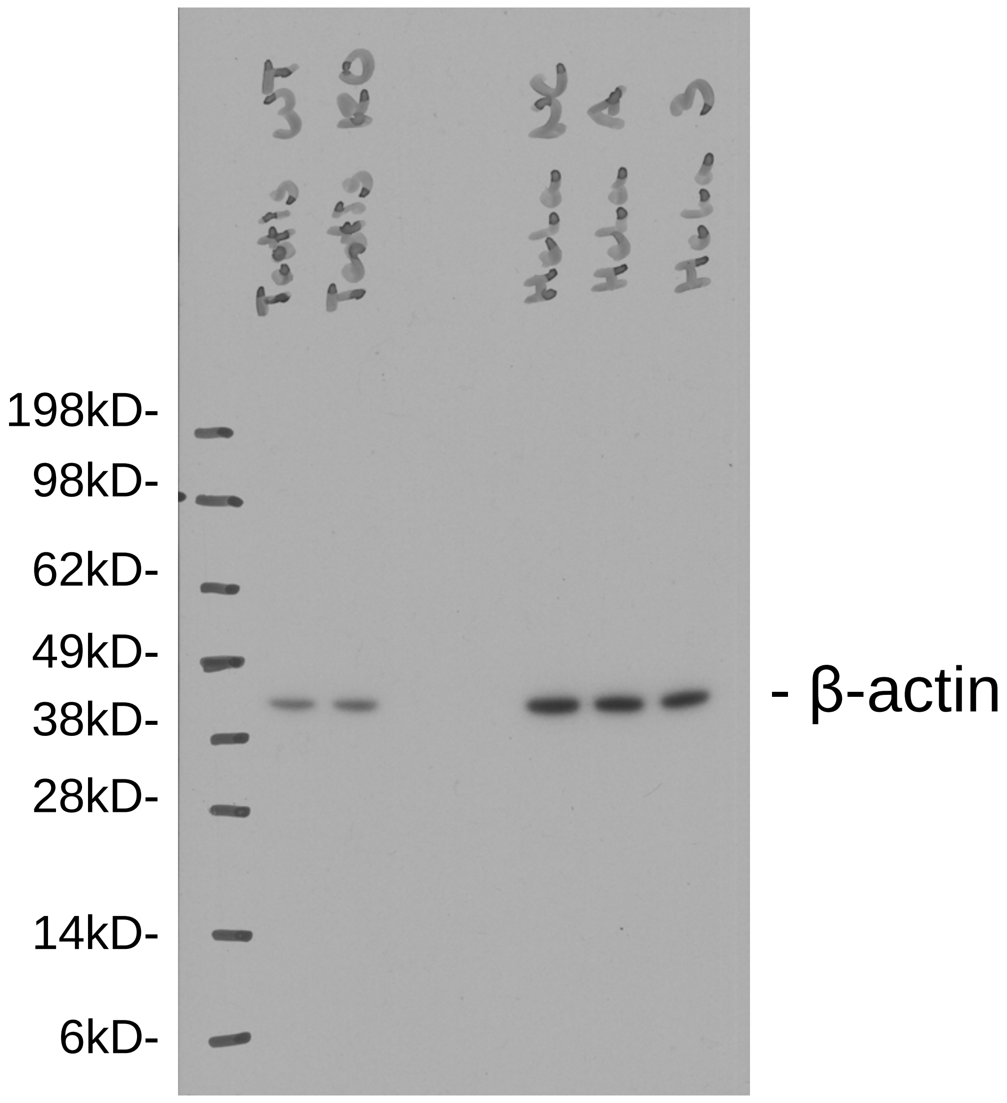

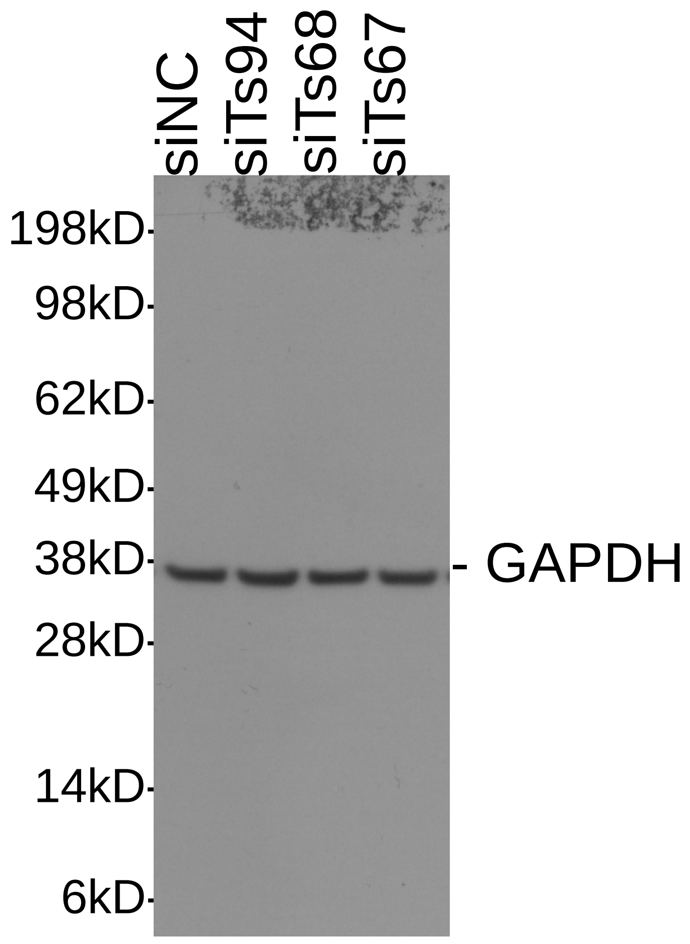

Uncropped images of immunoblots presented in Supplementary Figure S2 and S3.
